# Taxonomic and Functional Skin Microbiome Profiles in Nummular Eczema, Atopic Dermatitis, and Mixed Phenotypes: An Exploratory Shotgun Metagenomic Pilot Study

**DOI:** 10.3390/microorganisms14081631

**Published:** 2026-07-27

**Authors:** Mingyu Kim, You Kyung Koh, Seong Joo Kim, Kyung Hyun Min, Hyun Jeong Ju, Minho Lee, Young Bok Lee

**Affiliations:** 1 Department of Dermatology, Uijeongbu St. Mary’s Hospital, The Catholic University of Korea, Uijeongbu 11765, Republic of Korea; 2 Department of Industrial Pharmacy, Dongguk University, Seoul 04620, Republic of Koreaseongjoo@dgu.ac.kr (S.J.K.); 3 Department of Dermatology, St. Vincent’s Hospital, College of Medicine, The Catholic University of Korea, Suwon 16247, Republic of Korea; 4 Department of Life Science, Dongguk University, Goyang 10326, Republic of Korea

**Keywords:** nummular eczema, atopic dermatitis, skin microbiome, shotgun metagenomics, eczema

## Abstract

Nummular eczema (NE) and atopic dermatitis (AD) share clinical features, yet their skin microbiomes remain insufficiently characterized. This exploratory pilot study evaluated the taxonomic composition and functional potential of the skin microbiome across NE, a mixed NE/AD phenotype (Mixed NE_AD), and AD using shotgun metagenomics. Lesional swabs from Korean patients with NE (*n* = 8), Mixed NE_AD (*n* = 6), and AD (*n* = 5) were sequenced to assess microbial diversity, taxonomy, and functional pathways. *Staphylococcus aureus* predominated across all groups. NE samples were characterized by the higher relative abundance of environmental taxa, wherein *Delftia acidovorans* was observed only in NE samples in this cohort, and *Kocuria palustris* diminished sequentially across the Mixed NE_AD and AD phenotypes. Mixed NE_AD exhibited greater intra-genus *Staphylococcus* diversity. Despite non-significant overall diversity differences, partial ecological separation was noted. Functionally, descriptive differences in metabolic potential were observed, with NE showing trends toward biosynthetic and oxidative stress-associated pathways and AD showing trends toward degradation-related pathways. Mixed NE_AD showed intermediate characteristics. Although these differences did not reach statistical significance after correction for multiple testing, these findings provide preliminary evidence for microbiome-based stratification of eczematous disorders, warranting larger cohort validation.

## 1. Introduction

Nummular eczema (NE) is a chronic inflammatory skin disorder characterized by well-demarcated, coin-shaped and pruritic eczematous plaques [1]. The clinical course is often marked by persistent oozing and recurrent flares, predominantly localized to the lower extremities [2]. Due to its chronic and treatment-refractory nature, NE imposes a significant impairment on patients’ psychological well-being, social functioning, and overall quality of life [3]. Despite its clinical prevalence, the immunologic and microbial mechanisms underlying NE remain poorly understood [4].

NE is traditionally considered as an endogenous or idiopathic form of eczema associated with epidermal barrier disruption, immune activation, and microbial colonization [5]. Its relationship to atopic dermatitis (AD) remains a subject of debate; however, recent transcriptomic analysis showed that NE was upregulated with both type 2 and type 3 immune signatures, suggesting they are distinct entities [6,7].

These immunologic differences raise the possibility that NE may harbor a unique microbial ecosystem compared with AD.

Host–microbial crosstalk is a fundamental regulator of cutaneous immune responses. While microbial dysbiosis, specifically *Staphylococcus* aureus colonization and reduced microbial diversity, is well documented in AD, the skin microbiome of NE has yet to be comprehensively explored [8,9,10,11].

In this pilot exploratory study, we aimed to clarify whether these clinically overlapping disorders represent differential microbiome trends or exist along a shared spectrum by performing a comparative metagenomic analysis of NE and AD.

## 2. Materials and Methods

### 2.1. Study Population and Sample Collection

We conducted a cross-sectional study from June 2022 to October 2023 at the outpatient dermatology clinic at Uijeongbu St. Mary’s Hospital, Korea. A total of 19 patients were included in this study. The diagnosis of AD was established according to the Hanifin and Rajka criteria. NE was diagnosed clinically by board-certified dermatologists based on characteristic coin-shaped eczematous plaques. The study population was categorized into three groups: the NE group (*n* = 8) defined as patients newly diagnosed with NE without a prior history of AD, Mixed NE_AD group (*n* = 6) defined as patients with a confirmed history of AD and superimposed nummular lesions, and AD group (*n* = 5) where patients presented with typical AD features. The patient’s age, sex, disease severity according to the Eczema Area and Severity Index (EASI) score, and previous/current treatments were collected. All samples were obtained from the most recent active lesional skin on the lower extremities to minimize anatomical site variability. In the AD group, ongoing use of immunomodulatory agents (e.g., cyclosporine, methotrexate) or topical therapies (topical corticosteroids or calcineurin inhibitors) was not restricted, as the study aimed to reflect real-world clinical conditions. Although ongoing immunomodulatory treatment was not discontinued, this design allowed for evaluation of microbiome profiles in clinically relevant disease states, while acknowledging the potential influence of background therapy on microbial composition.

Skin microbiome samples were collected from active eczematous lesions on the lower extremities using EO gas-sterilized flocked swabs pre-moistened with sterile 0.9% saline. The lower extremities were selected because they were affected in all participants, thereby minimizing anatomical site variation across disease groups. The swab was gently rubbed across the lesional surface to collect superficial microbial communities. The skin was not disinfected before sampling to preserve the resident skin microbiota. Although the duration and number of swab strokes were not predefined, all samples were collected by trained dermatologists using the same procedure. After collection, the swabs were immediately placed into sterile empty collection tubes without transport medium and stored at 4 °C until DNA extraction. Swab-based sampling was selected because it is a minimally invasive, reproducible, and widely used approach for skin microbiome studies [12,13]. This study was approved by the Institutional Review Board of Uijeongbu St. Mary’s Hospital (XC22SIDI0067). Written informed consent was obtained from all participants for the collection of samples and publication of their clinical data.

### 2.2. Shotgun Metagenomic Sequencing and Quality Control

Genomic DNA quality was assessed using a spectrophotometer (Infinium F-200, NanoDrop; Thermo Fisher Scientific, Waltham, MA, USA), with acceptable samples showing an A260/A280 ratio of 1.6–2.0 and an A260/A230 ratio greater than 1.6. DNA concentration was quantified using a Qubit fluorometer (Thermo Fisher Scientific, Waltham, MA, USA) with dsDNA-binding dye chemistry, and integrity was verified by automated electrophoresis (4200 Tapestation; Agilent Technologies, Santa Clara, CA, USA).

Library preparation was carried out using the TruSeq DNA PCR-Free Library Preparation Kit (Illumina, San Diego, CA, USA). Library size distribution and quality were checked with the Agilent 2100 Bioanalyzer (Agilent Technologies, Santa Clara, CA, USA). We utilized the Illumina NovaSeq 6000 (Illumina, San Diego, CA, USA) system to obtain paired-end reads (2 × 150 bp). Accuracy was validated with >90% of bases achieving a Phred quality score of 30 (Q30).

Initially, sequencing quality was evaluated using FastQC (v0.11.9) and Sickle (v1.33). Trimmed reads were first aligned to the human reference genome (GRCh38) using BWA-MEM2 (v2.2.1) and human-derived reads were removed prior to downstream microbial analysis. The resulting SAM files were converted to BAM format and sorted using Samtools (v1.10) to optimize storage efficiency and access speed. Duplicate reads were removed to reduce potential amplification and sequencing bias. The proportion of host-derived reads was quantified for each sample to ensure adequate microbial sequencing depth.

### 2.3. Taxonomic and Functional Analysis

Taxonomic and functional profiles were analyzed to characterize the microbial communities. For taxonomic classification, Kraken2 (v2.1.2) was employed, utilizing k-mer-based alignment against a comprehensive database encompassing bacteria, archaea, viruses, and fungi. To enhance the accuracy of taxonomic abundance estimates, Bracken (v2.8) was applied, to provide refined relative abundance (total fraction reads) measurements. As the data were already represented as relative abundances, which inherently account for variations in sequencing depth, additional normalization steps such as rarefaction or conversion to Copies Per Million (CPM) were not performed to maintain the integrity of the total fraction profiles. Microbial diversity was assessed using these outputs. Alpha diversity, reflecting within-sample richness and evenness, was quantified using the Shannon index based on the relative abundance profiles. Beta diversity, which evaluates inter-sample variation in community composition, was analyzed using Non-metric Multidimensional Scaling (NMDS), which represents rank-based dissimilarities in community structure. Functional potential of the microbial communities was assessed using HUMAnN 3 (v3.0.0) from the BioBakery suite. Following the same principle as the taxonomic analysis, functional pathway abundances were analyzed using their respective relative abundance values (total fraction reads) to ensure consistency across all diversity and functional assessments.

### 2.4. Statistical Analysis

Microbiome taxonomic profiles were analyzed using the phyloseq (version 1.48.0) and vegan (version 2.6-6.1) packages. For taxonomic diversity analysis, the Shannon index was calculated at the phylum, genus, and species levels. Alpha diversity (Shannon index) and functional pathway abundance were compared among groups (NE, Mixed NE_AD, and AD) using the Kruskal–Wallis test, followed by Dunn’s test for post hoc pairwise comparisons where applicable. Differences in relative abundances of specific taxa at the species level among the groups were evaluated using the Kruskal–Wallis test followed by Dunn’s test with FDR correction. For low-abundance taxa evaluated primarily by detection frequency, including *Delftia acidovorans* and *Kocuria palustris*, presence/absence across groups was assessed using Fisher’s exact test. These analyses were considered exploratory.

The statistical significance of beta diversity was assessed using Permutational Multivariate Analysis of Variance (PERMANOVA) implemented with the adonis2 function in the vegan package (999 permutations). Correlation between the microbial abundance and clinical severity (EASI score) was evaluated using Spearman’s rank correlation analysis with FDR correction to identify level-specific associations. To account for multiple testing, *p*-values were adjusted using the Benjamini–Hochberg false discovery rate (FDR) method. A *p*-value (or q-value, where applicable) < 0.05 was considered statistically significant. All statistical analyses were performed using R software (version 4.5.1; R Foundation for Statistical Computing, Vienna, Austria).

## 3. Results

### 3.1. Demographics of the Study Population

A total of 19 patients were enrolled, comprising 8 patients with NE, 6 patients with Mixed NE_AD, and 5 patients with AD. The mean age of patients was 37.75 ± 22.56 years in the NE group, 31.83 ± 13.07 years in the Mixed NE_AD group, and 32.20 ± 14.22 years in the AD group. Disease severity, assessed via EASI score at the time of sampling, showed mean values of 9.66 ± 6.15 (NE), 11.57 ± 3.52 (Mixed NE_AD), and 25.10 ± 11.57 (AD) (Table 1). The AD group exhibited higher EASI scores compared to the NE and NE_AD groups. Although this difference did not reach formal statistical significance, a strong trend was observed among the groups (Kruskal–Wallis test, *p* = 0.062). Post hoc pairwise comparisons with Benjamini–Hochberg adjustment revealed no statistically significant differences between individual groups (AD vs. NE, *p* = 0.10; AD vs. NE_AD, *p* = 0.18). Correlation analysis was performed to evaluate the relationship between diversity and EASI score within each group to adjust for its potential influence on microbial richness and evenness.

### 3.2. Microbial Signatures of Nummular Eczema and Atopic Dermatitis

Shotgun metagenomic profiling revealed a divergence in community structure from the phylum to species level across the three groups.

**Figure 1:**
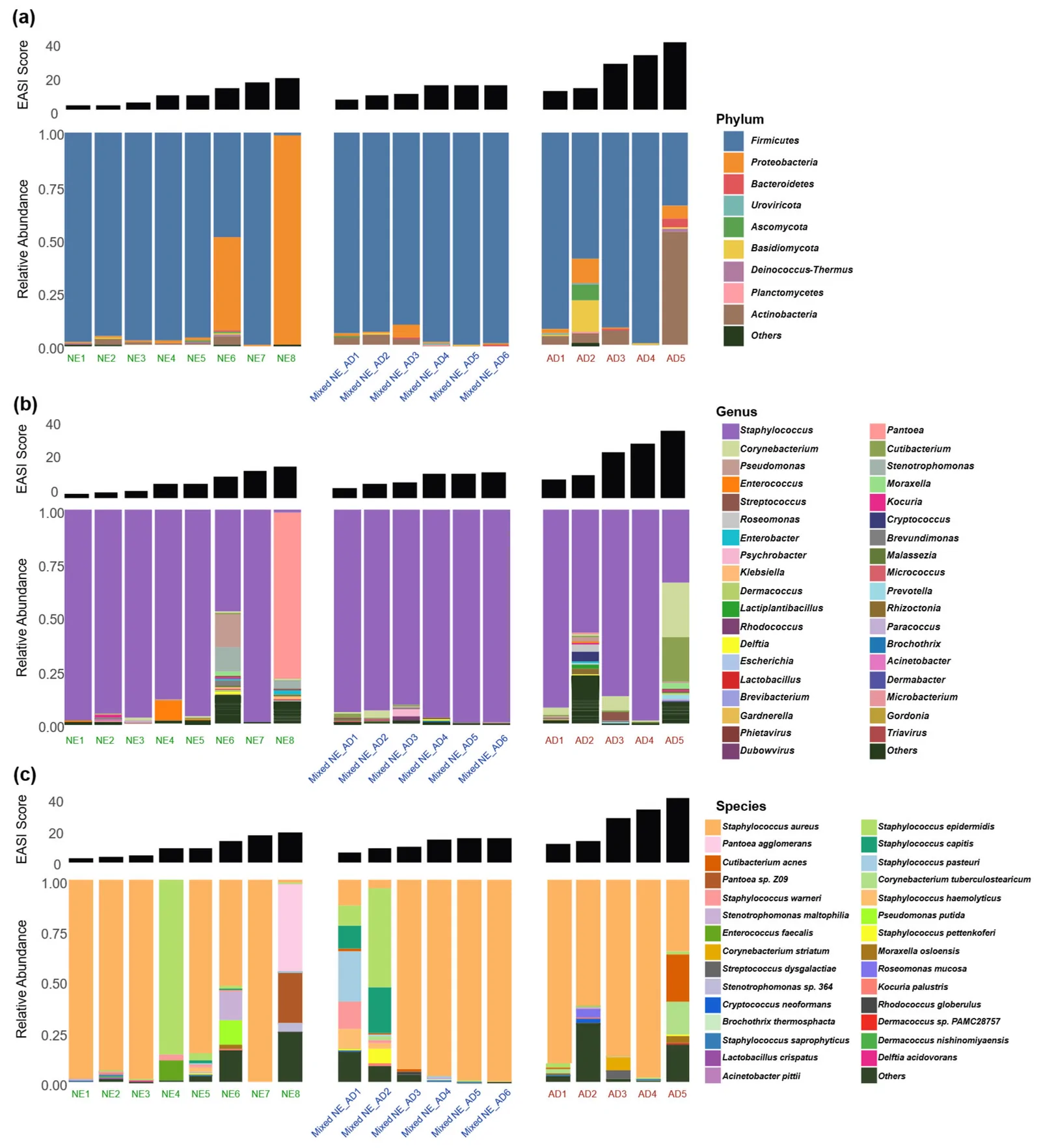
Microbial composition by taxonomic level and EASI score. Stacked bar plots show the relative abundance of microbial taxa at the phylum (**a**), genus (**b**), and species (**c**) levels across all skin samples. Samples are grouped by clinical condition—nummular eczema (NE), overlapping NE and atopic dermatitis (Mixed NE_AD), and atopic dermatitis (AD)—and are ordered from left to right according to increasing EASI score. Black bars above each column represent individual EASI scores.

At the phylum level (Figure 1a), the NE and Mixed NE_AD groups were characterized by a predominance of *Firmicutes*, whereas the AD group exhibited a pronounced shift toward *Actinobacteria* and *Proteobacteria*. This taxonomic shift was most pronounced in AD samples with high EASI scores (e.g., AD4 and AD5). Genus-level analysis (Figure 1b) indicated that *Staphylococcus* was dominant across all groups. However, *Corynebacterium* was preferentially enriched in AD, while NE samples (e.g., NE6) showed a higher relative abundance of *Stenotrophomonas*, an environmental organism often associated with compromised skin barriers. At the species level (Figure 1c), *Staphylococcus epidermidis* and *Staphylococcus aureus* were present in both NE and AD, while *Stenotrophomonas maltophilia* and *Corynebacterium kroppenstedtii* appeared more frequently in NE. In contrast, *Corynebacterium striatum* and *Cryptococcus neoformans* were predominantly detected in AD.

**Figure 2:**
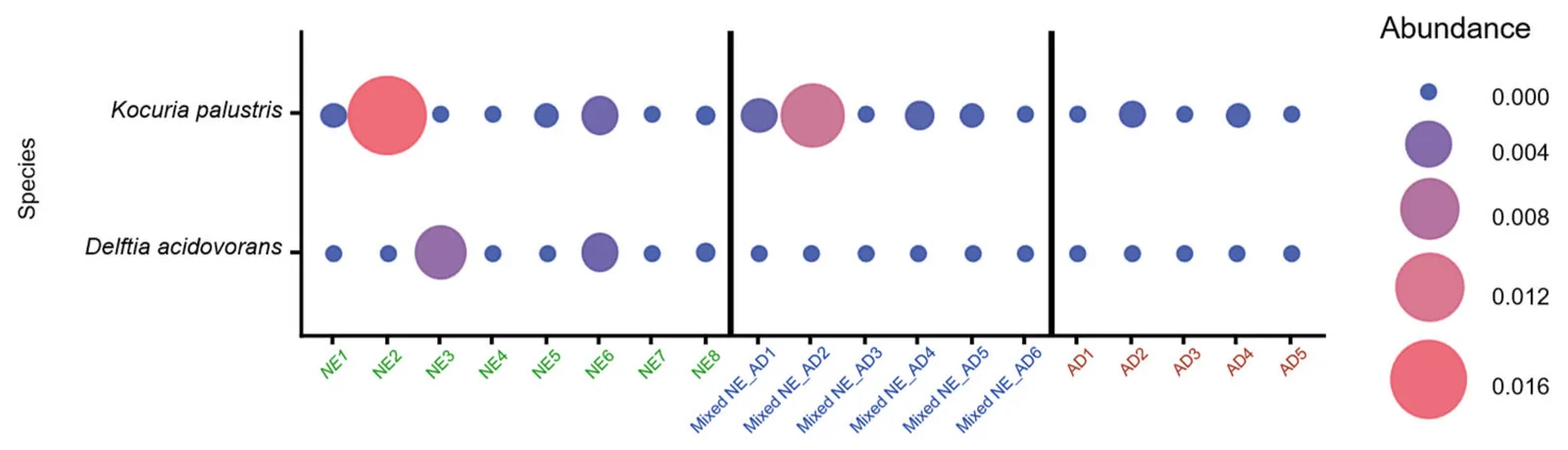
Relative abundance of *Delftia acidovorans* and *Kocuria palustris* across clinical groups. Dot plot visualizing the relative abundance of two species, *Delftia acidovorans* and *Kocuria palustris*, across NE, Mixed NE_AD, and AD groups. *Delftia acidovorans* was observed only in NE samples in this cohort, whereas *Kocuria palustris* showed a decreasing detection pattern from NE to Mixed NE_AD and AD. Dot size and color intensity correspond to species abundance per sample.

The low-abundance taxa, *Delftia acidovorans* and *Kocuria palustris* were predominantly detected in NE samples, but their distribution profiles across the other phenotypes differed. Specifically, *Delftia acidovorans* was exclusively detected in NE samples and completely absent in both the Mixed NE_AD and AD groups. Conversely, *Kocuria palustris* demonstrated a graded presence, decreasing from NE to Mixed NE_AD and AD (Figure 2). The presence of these environmental organisms primarily in NE samples within this cohort may suggest an increased susceptibility to external microbial infiltration, potentially compatible with a barrier-compromised pathogenic mechanism.

### 3.3. Microbial Diversity and Correlation with Disease Severity

**Figure 3:**
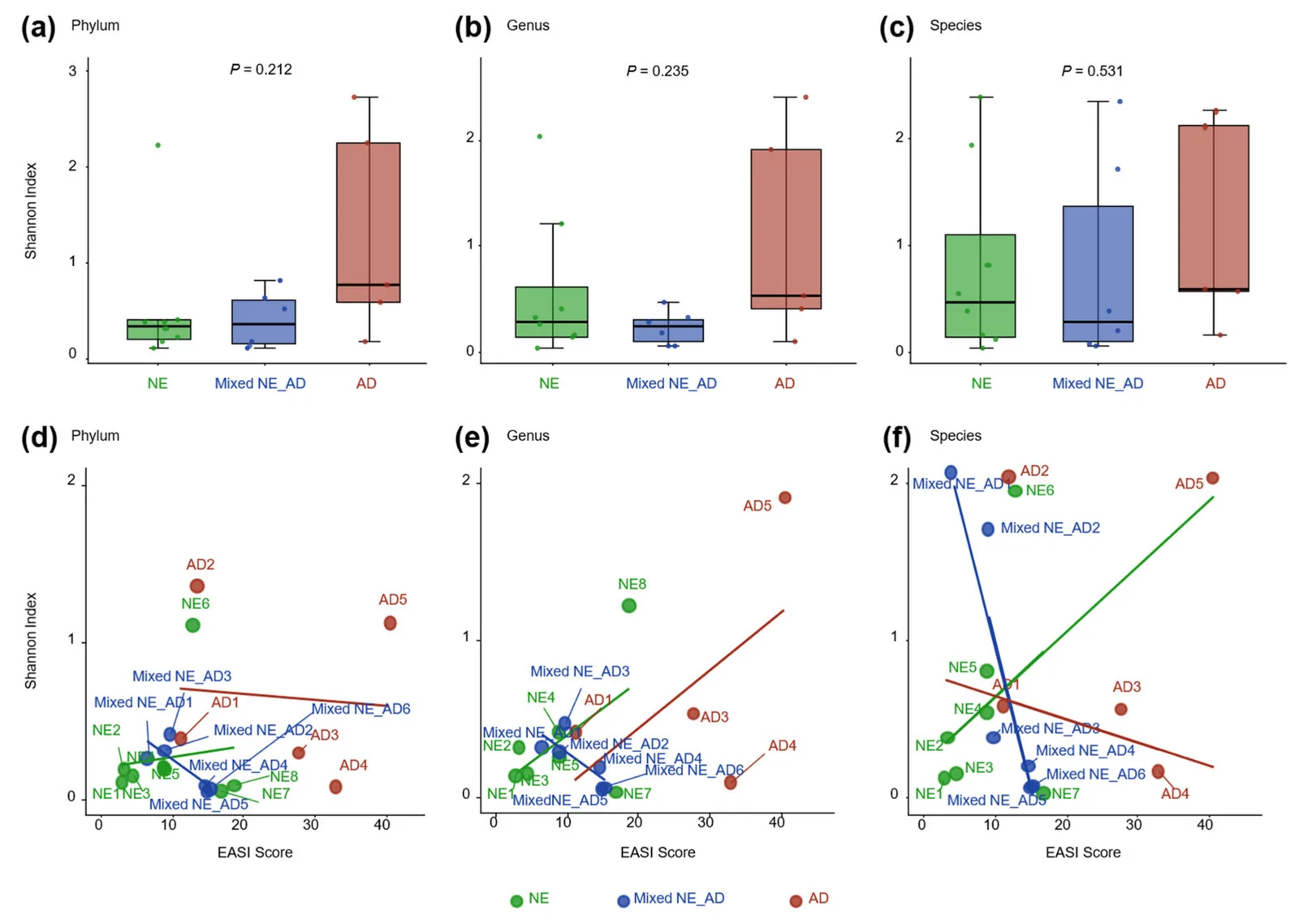
Microbial diversity and its correlation with clinical severity. Boxplots represent the Shannon index at the phylum (**a**), genus (**b**), and species (**c**) levels across NE, Mixed NE_AD, and AD groups. *p*-values (**a**–**c**) were determined using the Kruskal–Wallis test across the three groups. Scatterplots show the relationship between the Shannon index and EASI score for each group at the phylum (**d**), genus (**e**), and species (**f**) levels.

Alpha diversity, measured by the Shannon index, revealed no statistically significant difference among the NE, Mixed NE_AD, and AD groups at the phylum, genus, or species levels (all *p* > 0.05), though the AD group tended to exhibit slightly higher species-level diversity (Figure 3a–c). Correlation analysis revealed no significant relationship between Shannon diversity and EASI scores at the phylum or genus levels across groups (Figure 3d,e). However, at the species level, a significant negative correlation was observed in the Mixed NE_AD group (*p* = 0.017), while an opposite (positive) trend was observed in the NE group (Figure 3f). These findings suggest that an increased inflammatory burden could be associated with a reduction in microbial species diversity.

Beta diversity analysis using NMDS ordination showed a visual pattern compatible with partial group separation; however, this pattern did not reach statistical significance in PERMANOVA, including the covariate-adjusted model (Table S1). Samples showed partial separation by diagnostic group, but were not clustered by age or sex (Figure S1). NE and AD occupied divergent positions in ordination space, whereas Mixed NE_AD samples were consistently positioned between them. Although this separation did not reach statistical significance (*p* > 0.05), the spatial pattern indicates that the microbial signature of the Mixed NE_AD group characterizes an overlapping phenotype, where the co-occurrence of AD and NE creates a unique niche that harbors shared taxonomic features of both primary conditions.

### 3.4. Expanded Staphylococcus Species Diversity in the Mixed Phenotype

**Figure 4:**
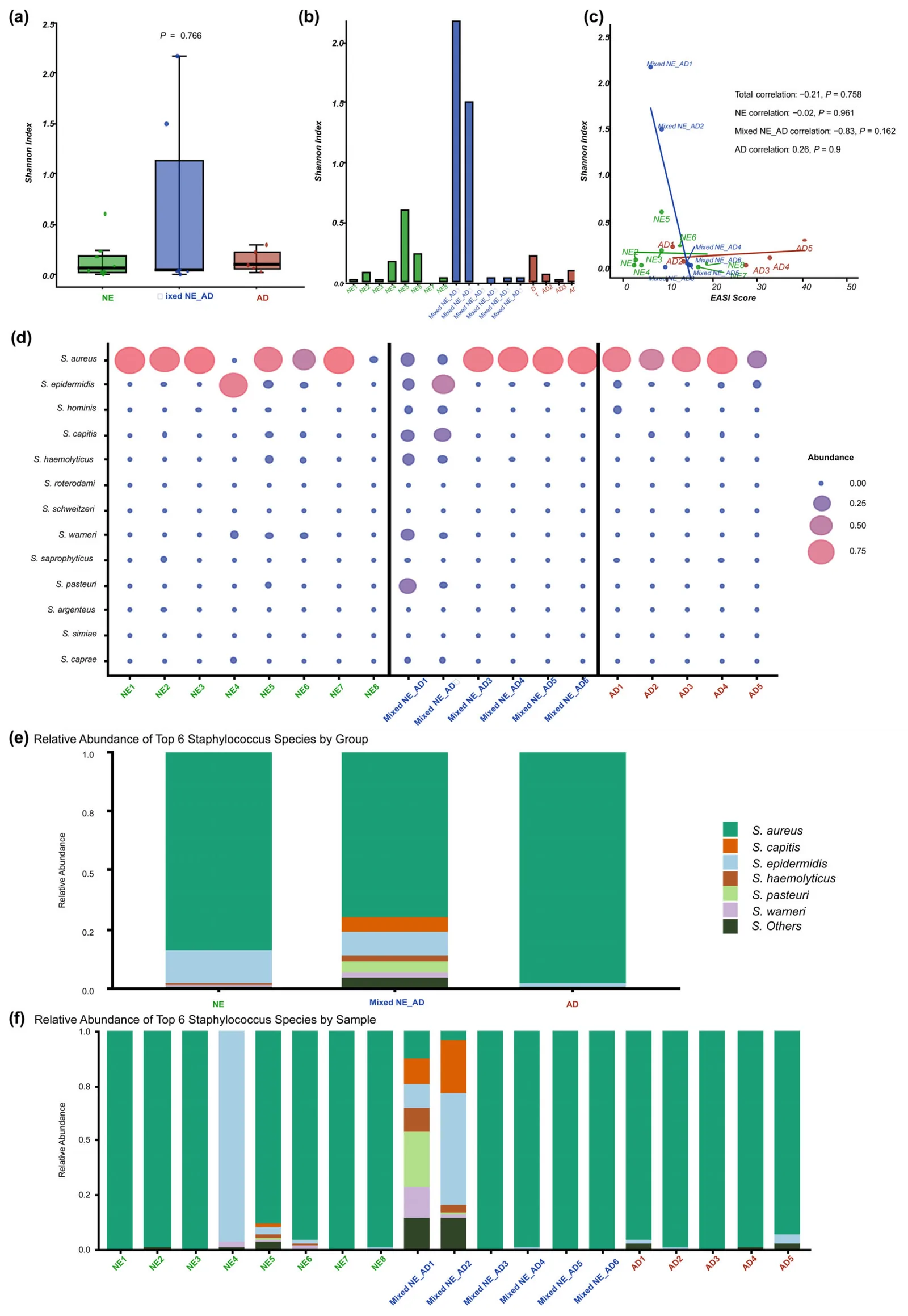
Intra-genus diversity, clinical correlation, and composition of *Staphylococcus*. (**a**) Shannon index of *Staphylococcus* species across clinical groups. *p*-value was calculated using the Kruskal–Wallis test. (**b**) Shannon index for an individual sample. (**c**) Correlation between the Shannon index of *Staphylococcus* species and EASI score. (**d**) Dot plot depicting the presence and relative abundance of 13 *Staphylococcus* species across all samples. (**e**) Stacked bar plots showing the relative abundance of the top six *Staphylococcus* species at the group level. (**f**) Stacked bar plots of the top six species at the individual sample level.

Given the dominance of *Staphylococcus* across all samples, we further examined intra-genus dynamics. The Mixed NE_AD group exhibited a higher median Shannon diversity for *Staphylococcus* species, although statistically not significant (Kruskal–Wallis *p* = 0.235). Individual-level plots (Figure 4b) highlighted substantial inter-individual variability within the Mixed NE_AD group, suggesting that this group harbors a more diverse *Staphylococcus* community. In addition, a negative trend was observed between *Staphylococcus* diversity and EASI score within the Mixed NE_AD group; however, this association did not reach statistical significance after the revised analysis. (r = −0.83, *p* = 0.162) (Figure 4c).

*S. aureus* remained the predominant species among 13 *Staphylococcus* species across nearly all samples (Figure 4d). However, Mixed NE_AD cases displayed a marked increase in non-aureus taxa, including commensals such as *S. epidermidis*, *S. hominis*, and *S. capitis*, as well as less frequently detected organisms like *S. pasteuri*, *S. haemolyticus*, and *S. warneri*. This broader species repertoire was less apparent in the pure NE or AD group, where *S. aureus* tended to exhibit stronger dominance (Figure 4e,f).

### 3.5. Functional Pathway Shifts Among Groups

**Figure 5:**
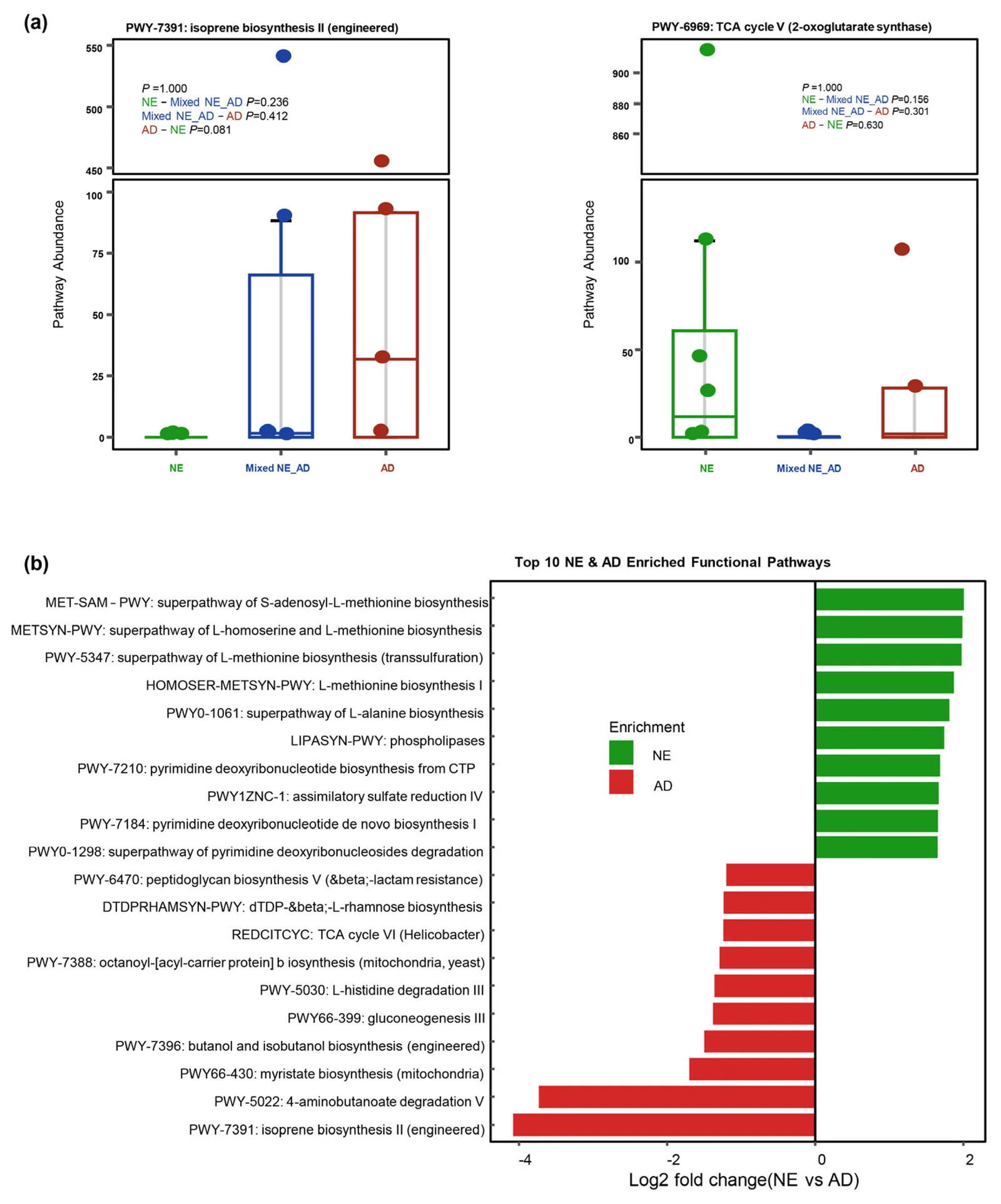
Differentially abundant microbial pathways among groups. (**a**) Boxplots display the relative abundance of two functional pathways across the groups (NE, Mixed NE_AD, AD). *p*-values were calculated using the Kruskal–Wallis test, followed by Dunn’s test for post hoc pairwise comparisons. All *p*-values were adjusted for multiple comparisons using the Benjamini–Hochberg (FDR) method. None of the functional pathways reached statistical significance (*p* > 0.05) after FDR correction. (**b**) Top 10 functional pathways enriched in the NE and AD groups, respectively. Pathways are ranked based on the Log2 fold change (NE vs. AD), showing the most differentially abundant pathways between the two groups.

Functional profiling suggested descriptive differences in metabolic potential among the groups, although none of the tested pathways reached statistical significance after FDR correction (Figure 5). NE samples demonstrated consistent enrichment of ‘biosynthetic and oxidative stress-responsive pathways, including methionine biosynthesis’ (PWY-5347), the ‘γ-glutamyl cycle’ (PWY-4041), and ‘alanine biosynthesis’ (PWY0-1061). In contrast, AD samples showed greater abundance of degradation-oriented pathways, particularly those related to amino acid catabolism and proteolytic activity. Mixed NE_AD samples exhibited an intermediate profile showing enrichment of both biosynthetic (NE-like) and degradative (AD-like) pathways.

## 4. Discussion

This study presents the first high-resolution shotgun metagenomic characterization comparing the skin microbiomes of NE, Mixed NE_AD, and AD. While all groups were dominated by *Staphylococcus* species, we identified potential taxonomic variations, which may suggest differing microbial associations with NE and AD. Notably, the Mixed NE_AD group manifested intermediate microbial features, which is compatible with its clinical characteristics as an overlapping phenotype; however, the biological significance of these trends requires further validation.

All groups were dominated by *Staphylococcus* species, particularly *S. aureus*, a well-recognized hallmark of AD-associated dysbiosis that correlates with disease severity and type II immune biomarkers, such as eosinophil count, total IgE, CCL17, and periostin [14,15]. In NE lesions, however, we observed higher relative abundance of unique environmental taxa, including *Stenotrophomonas maltophilia*, *Delftia acidovorans* (*D. acidovorans*), and *Kocuria palustris*.

While *Kocuria* and *Delftia* are often considered opportunistic pathogens in immunocompromised hosts, their detection in a subset of NE samples might merely reflect transient presence from the environment due to an impaired physical skin barrier [16,17,18]. *Kocuria* species are skin commensals with broad environmental distribution, and are an emerging opportunistic pathogen in immunocompromised patients [19]. Known for its role in metal homeostasis and biofilm formation, *D. acidovorans* has been reported to influence the growth of Gram-positive bacteria through the secretion of delftibactin and other metabolites [11,19,20,21].

Several studies suggest that *D. acidovorans*-derived metabolites can inhibit the growth of *S. epidermidis* via TCA cycle-mediated reactive oxygen species production, [22,23] potentially facilitating the overgrowth of *S. aureus* [24,25,26].

Consistent with these findings, our functional profiling showed an increasing trend of the TCA cycle-related pathway (PWY-6969) in NE samples. The presence of these atypical, environmental organisms, together with trends in inferred functional pathways, may be compatible with altered barrier-associated microbial exposure in NE.

Functional profiling further delineated the metabolic trends of NE and AD. NE lesions were more frequently detected in biosynthetic pathways, including methionine and alanine biosynthesis, and oxidative stress markers, including the TCA cycle-related pathway (PWY-6969). Prior studies have shown that TCA cycle activation in certain bacteria can enhance resilience to oxidative stress [27]. The concurrent enrichment of biosynthetic and oxidative stress-responsive pathways in NE samples may be linked to microbial redox regulation and amino acid replenishment, suggesting that the NE-associated microbiome may be adapting to a barrier-compromised, highly oxidative microenvironment. Importantly, these functional implications are derived from inferred metabolic pathways, as shotgun metagenomics estimates functional potential rather than actual metabolic activity. Validation through transcriptomic, metabolomic, or experimental approaches is necessary before drawing definitive mechanistic conclusions.

In contrast, AD lesions exhibited a predominance of degradation-associated pathways, especially amino acid catabolism and proteolytic activity. This functional profile aligns with the virulence factors of *S. aureus*, which produces extracellular proteases such as staphylococcal serine protease (V8 protease; SspA), cysteine protease (SspB), metalloprotease (aureolysin; Aur), and staphopain (Scp) that further degrade the skin barrier and exacerbate immune dysregulation [28,29,30]. These contrasting metabolic profiles may suggest differences in the microbial ecology underlying these two conditions. These functional profiles indicate a descriptive trend of contrasting metabolic potentials between the conditions. Importantly, these functional implications are derived from inferred metabolic pathways, as shotgun metagenomics estimates functional potential rather than actual metabolic activity. Validation through transcriptomic, metabolomic, or experimental approaches would be necessary before drawing definitive mechanistic conclusions regarding barrier degradation or immune dysregulation.

The Mixed NE_AD group demonstrated hybrid taxonomic and functional profiles, including increased *Staphylococcus* species diversity and moderate representation of biosynthetic and degradative pathways. These findings support that the Mixed NE_AD may reflect a transitional state, reflecting shared features of both NE and AD. The spatial positioning of Mixed NE_AD between NE and AD in our diversity analysis is compatible with the concept of a continuous disease spectrum, though this requires validation in larger cohorts.

Several limitations should be noted. First, this study was conducted as an exploratory pilot study with an extremely small and heterogeneous cohort (eight NE, six Mixed NE_AD, and five AD patients). Because this study did not include a healthy control cohort, our findings are best interpreted in the context of previously published healthy skin microbiome data. In healthy skin, moist and sebaceous sites are typically dominated by *Cutibacterium* and *Corynebacterium*, with commensal *Staphylococcus* species such as *S. epidermidis* generally predominating over *S. aureus*, reflecting a state of microbial homeostasis [31]. Against this baseline, the *S. aureus*-dominated, comparatively low-diversity communities observed across all three groups in our cohort are consistent with a dysbiotic shift shared by eczematous skin, upon which the more subtle, phenotype-associated differences described here appear to be superimposed. In the absence of an age- and site-matched healthy control arm, however, our design cannot formally distinguish disease-specific signatures from this generic inflamed-skin dysbiosis, and direct comparison with healthy controls should be a priority for future studies. This limited sample size, combined with the loss of statistical significance for most comparisons after multiple testing correction, means our findings primarily represent descriptive trends rather than definitive conclusions. Second, the cross-sectional design provides only a snapshot of the microbiome, preventing assessment of temporal dynamics or causal relationships. Third, while shotgun metagenomics offers high-resolution taxonomic and functional data, functional predictions are limited by database completeness and cannot directly measure active gene expression or metabolic activity. Lastly, substantial confounding variables, notably differences in disease severity and ongoing immunomodulatory treatments, were not adequately controlled or incorporated into the statistical analyses. Consequently, it is difficult to determine whether the observed microbial differences truly reflect descriptive trends of diseases or merely represent treatment- or severity-associated variations.

Despite these limitations, our pilot study provides the first high-resolution taxonomic and functional characterization of NE and its relationship with AD. While these descriptive trends provide a preliminary understanding of the taxonomic and functional differences between NE and AD, future studies incorporating larger, well-controlled cohorts and functional multi-omics are required to validate these findings and elucidate their true clinical significance.

## Figures and Tables

**Table 1:** Clinical characteristics and current treatments of participants with NE, Mixed NE_AD, and AD.

Group	ID	Age	Sex	EASI Score	Current Treatment
NE	NE1	25	F	2.8	Cs
	NE2	19	F	3.2	TCS
	NE3	19	M	4.4	TCS
	NE4	23	F	8.7	SCS
	NE5	69	M	8.8	SCS, Cs, TCS
	NE6	74	M	12.8	Cs, TCS
	NE7	52	F	16.7	TCS
	NE8	21	M	18.7	SCS, TCS
Mean ± SD		37.8 ± 22.6		9.7 ± 6.2	
Mixed NE_AD	Mixed NE_AD1	28	F	6.4	Cs, TCS
	Mixed NE_AD2	19	F	8.9	SCS, TCS
	Mixed NE_AD3	38	F	9.6	Cs, TCS
	Mixed NE_AD4	49	F	14.5	SCS, TCS
	Mixed NE_AD5	7	M	14.8	SCS, TCS
	Mixed NE_AD6	39	F	15.2	SCS, TCS
Mean ± SD		31.8 ± 13.1		11.6 ± 3.5	
AD	AD1	54	M	11.1	Cs, TCS
	AD2	22	F	13.5	TCS
	AD3	46	M	27.6	MTX, TCI
	AD4	23	M	32.8	Cs, TCS
	AD5	16	M	40.5	SCS, TCS
Mean ± SD		32.2 ± 14.2		25.1 ± 11.6	

Abbreviations: SCS: Systemic Corticosteroid; Cs: Cyclosporine; MTX: Methotrexate; TCS: Topical Corticosteroid; TCI: Topical Calcineurin Inhibitor.

## Data Availability

The datasets presented in this article are not readily available because they contain patient-derived shotgun metagenomic sequencing data and associated clinical metadata from a small clinical cohort, and public release is restricted by participant privacy considerations, informed-consent limitations, and institutional ethics requirements. Requests to access the datasets should be directed to the corresponding authors (lyb80@catholic.ac.kr (Y.B.L.); minholee@dgu.edu (M.L.)). Access may be granted upon reasonable request, subject to institutional review and applicable data-sharing agreements.

## Supplementary Materials

The following supporting information can be downloaded at https://www.mdpi.com/article/10.3390/microorganisms14081631/s1, Figure S1: Beta diversity analysis by demographic and clinical variables; Table S1. Covariate-adjusted PERMANOVA evaluating the influence of clinical parameters and treatments on microbial community composition; Table S2. Comparison of alpha diversity indices and effect sizes among the clinical groups; Table S3. Comparison of *Staphylococcus* species diversity and effect size among the clinical groups. Table S4. Detection frequencies of *Delftia acidovorans and Kocuria palustris* among the clinical groups; Table S5. Comparison of taxonomic relative abundances at the species level among the clinical groups; Table S6. Post hoc pairwise analysis of bacterial relative abundances with Benjamini–Hochberg FDR correction; Table S7. Comparison of functional pathway abundances and effect sizes among the clinical groups.

## Abbreviations

The following abbreviations are used in this manuscript:

NE	Nummular eczema
Mixed NE_AD	Mixed nummular eczema and atopic dermatitis phenotype
AD	Atopic dermatitis
Cs	Cyclosporine
TCS	Topical corticosteroid
SCS	Systemic corticosteroid
MTX	Methotrexate
TCI	Topical calcineurin inhibitor
IQR	Interquartile range
