# Supplementary material for: Taxonomic and Functional Skin Microbiome Profiles in Nummular Eczema, Atopic Dermatitis, and Mixed Phenotypes: An Exploratory Shotgun Metagenomic Pilot Study
Source: Microorganisms. 2026 Jul 27;14(8):1631. doi: 10.3390/microorganisms14081631 (PMC13515936; doi:10.3390/microorganisms14081631)
Supplement: Supplementary file 1 [file microorganisms-14-01631-s001.zip › microorganisms-4409137-supplementary.pdf]

Table S1. Covariate-adjusted PERMANOVA evaluating the influence of clinical parameters and treatments on microbial community composition.

Variable	Dimension of freedom	Sum Of Sqs	R^2	F	Pr(>F)
EASI	1	0.094	0.026	0.409	0.813
Use_CS	1	0.053	0.015	0.231	0.950
Use_TCS	1	0.184	0.051	0.802	0.533
Use_SCS	1	0.169	0.047	0.735	0.496
Use_MTX	1	0.095	0.026	0.414	0.760
Group	2	0.203	0.056	0.440	0.918
Residual	11	2.530	0.701	NA	NA
Total	18	3.610	1.000	NA	NA

The table displays the results of a marginal permutational multivariate analysis of variance (PERMANOVA) based on Bray-Curtis dissimilarities with 999 permutations. This multivariate model was designed to evaluate the independent contribution of the disease group to the skin microbiome variation, while strictly controlling for the confounding effects of disease severity (EASI score) and concurrent immunomodulatory treatments. All values are rounded to three decimal places.

Table S2. Comparison of alpha diversity indices and effect sizes among the clinical groups.

Level	Effect size(ϵ^2)	Magnitude	NE (Median [IQR])	Mixed NE_AD (Median [IQR])	AD (Median [IQR])
Phylum	0.069	moderate	0.17 [0.10-0.20]	0.18 [0.07-0.30]	0.38 [0.29-1.12]
Genus	0.056	small	0.29 [0.15-0.62]	0.24 [0.10-0.32]	0.54 [0.42-1.91]
Species	0	negligible	0.47 [0.15-1.10]	0.30 [0.11-1.38]	0.59 [0.57-2.12]

Data are presented as median [IQR]. All values are rounded to three decimal places. The effect size (ϵ^2) was calculated using the Kruskal-Wallis test. Negative calculated adjusted effect sizes were constrained to 0, indicating a negligible effect.

Table S3. Comparison of *Staphylococcus* species diversity and effect size among the clinical groups.

Metric	Effect size(ϵ^2)	Magnitude	NE (Median [IQR])	Mixed NE_AD (Median [IQR])	AD (Median [IQR])
Shannon	0	negligible	0.06 [0.02-0.19]	0.04 [0.03-1.13]	0.09 [0.06-0.22]

Data are presented as median [IQR]. All values are rounded to three decimal places. The effect size (ϵ^2) was calculated using the Kruskal-Wallis test. Negative calculated adjusted effect sizes were constrained to 0, indicating a negligible effect.

Table S4. Detection frequencies of *Delftia acidovorans* and *Kocuria palustris* among the clinical groups.

Species	Clinical Group	Presence (n)	Absence (n)	*P*-value
Delftia acidovorans	NE (n=8)	3	5	0.089
	Mixed NE_AD (n=6)	0	6	
	AD (n=5)	0	5	
Kocuria palustris	NE (n=8)	5	3	0.713
	Mixed NE_AD (n=6)	4	2	
	AD (n=5)	2	3	

All values are rounded to three decimal places.; *n*, number of participants

Table S5. Comparison of taxonomic relative abundances at the species level among the clinical groups.

Species	Statistic	n	df	*P*-value
Corynebacterium kroppenstedtii	1.375	19	2	0.503
Corynebacterium striatum	3.622	19	2	0.163
Cryptococcus neoformans	2.8	19	2	0.247
Delftia acidovorans	4.6	19	2	0.1
Kocuria palustris	1.660	19	2	0.436
Roseomonas mucosa	0.116	19	2	0.944
Staphylococcus aureus	0.306	19	2	0.858
Staphylococcus epidermidis	0.873	19	2	0.646

Stenotrophomonas maltophilia	2.389	19	2	0.303

All comparisons were performed using the Kruskal-Wallis test. Abbreviations: df, degrees of freedom; *n*, number of participants. All values are rounded to three decimal places.

Table S6. Post-hoc pairwise analysis of bacterial relative abundances with Benjamini-Hochberg FDR correction.

Species	group1(n)	group2(n)	statistic	p	p.adj	p.adj.signif
Corynebacterium kroppenstedtii	NE(8)	Mixed NE_AD(6)	-1.009	0.313	0.509	ns
	NE(8)	AD(5)	-0.956	0.339	0.509	ns
	Mixed NE_AD(6)	AD(5)	0.000	1.000	1.000	ns
Corynebacterium striatum	NE(8)	Mixed NE_AD(6)	-0.550	0.582	0.582	ns
	NE(8)	AD(5)	1.441	0.149	0.224	ns
	Mixed NE_AD(6)	AD(5)	1.848	0.065	0.194	ns
Cryptococcus neoformans	NE(8)	Mixed NE_AD(6)	0.000	1.000	1.000	ns
	NE(8)	AD(5)	1.529	0.126	0.225	ns
	Mixed NE_AD(6)	AD(5)	1.440	0.150	0.225	ns
Delftia acidovorans	NE(8)	Mixed NE_AD(6)	-1.845	0.065	0.121	ns
	NE(8)	AD(5)	-1.748	0.080	0.121	ns
	Mixed NE_AD(6)	AD(5)	0.000	1.000	1.000	ns
Kocuria palustris	NE(8)	Mixed NE_AD(6)	0.463	0.643	0.643	ns
	NE(8)	AD(5)	-0.911	0.362	0.544	ns
	Mixed NE_AD(6)	AD(5)	-1.270	0.204	0.544	ns

Roseomonas mucosa	NE(8)	Mixed NE_AD(6)	0.318	0.750	0.984	ns
	NE(8)	AD(5)	0.020	0.984	0.984	ns
	Mixed NE_AD(6)	AD(5)	-0.265	0.791	0.984	ns
Staphylococcus aureus	NE(8)	Mixed NE_AD(6)	0.411	0.681	0.864	ns
	NE(8)	AD(5)	-0.171	0.864	0.864	ns
	Mixed NE_AD(6)	AD(5)	-0.528	0.597	0.864	ns
Staphylococcus epidermidis	NE(8)	Mixed NE_AD(6)	0.569	0.569	0.728	ns
	NE(8)	AD(5)	0.908	0.364	0.728	ns
	Mixed NE_AD(6)	AD(5)	0.347	0.728	0.728	ns
Stenotrophomonas maltophilia	NE(8)	Mixed NE_AD(6)	-1.479	0.139	0.417	ns
	NE(8)	AD(5)	-1.011	0.312	0.468	ns
	Mixed NE_AD(6)	AD(5)	0.368	0.713	0.713	ns

Pairwise comparisons were performed using exploratory Dunn's post-hoc comparisons. P-values were adjusted using the Benjamini-Hochberg false discovery rate (FDR) correction method. ns, not significant.. All values are rounded to three decimal places.

Table S7. Comparison of functional pathway abundances and effect sizes among the clinical groups.

**Pathway**	**Effect size (ϵ^2)**	**Magnitude**	**NE (Median [IQR])**	**Mixed NE_AD (Median [IQR])**	**AD (Median [IQR])**
14DICHLORBENZDEG-PWY: 1,4-dichlorobenzene degradation	0.05	small	0.000 [0.000-0.000]	0.000 [0.000-0.000]	0.000 [0.000-0.000]
1CMET2-PWY: folate transformations III (E. coli)	0	negligible	1.045 [0.000-2.562]	1.888 [0.379-2.430]	1.884 [0.000-2.389]
ANAEROFRUCAT-PWY:	0	negligible	2.256 [0.000-2.709]	1.959 [0.344-2.792]	2.091 [1.346-2.674]

homolactic fermentation					
ANAGLYCOLYSIS-PWY: glycolysis III (from glucose)	0	negligible	2.429 [0.711-2.922]	2.277 [0.491-2.888]	2.205 [1.491-2.797]
ARG+POLYAMINE-SYN: superpathway of arginine and polyamine biosynthesis	0	negligible	0.000 [0.000-0.000]	0.000 [0.000-0.000]	0.000 [0.000-0.000]
ARGININE-SYN4-PWY: L-ornithine biosynthesis II	0	negligible	0.000 [0.000-0.283]	0.000 [0.000-0.000]	0.000 [0.000-0.000]
ARGSYN-PWY: L-arginine biosynthesis I (via L-ornithine)	0	negligible	2.371 [0.964-2.916]	2.127 [0.461-2.860]	2.269 [1.581-2.739]
ARGSYNBSUB-PWY: L-arginine biosynthesis II (acetyl cycle)	0	negligible	2.505 [1.183-2.941]	2.284 [1.463-3.035]	2.394 [1.619-2.851]
ARO-PWY: chorismate biosynthesis I	0	negligible	2.138 [0.000-2.757]	2.104 [0.424-2.823]	2.088 [1.438-2.523]
ASPASN-PWY: superpathway of L-aspartate and L-asparagine biosynthesis	0	negligible	0.000 [0.000-0.321]	0.000 [0.000-0.000]	0.000 [0.000-1.122]
BIOTIN-BIOSYNTHESIS-PWY: biotin biosynthesis I	0	negligible	0.000 [0.000-0.000]	0.000 [0.000-0.000]	0.000 [0.000-0.000]
BRANCHED-CHAIN-AA-SYN-PWY: superpathway of branched chain amino acid biosynthesis	0	negligible	2.558 [1.199-3.085]	2.289 [1.523-2.996]	2.355 [1.615-2.806]
CALVIN-PWY: Calvin-Benson-Bassham cycle	0	negligible	2.472 [0.944-2.955]	2.341 [1.642-3.065]	2.381 [1.446-3.002]
CITRULBIO-PWY: L-citrulline biosynthesis	0	negligible	2.273 [1.381-2.988]	2.225 [0.389-3.131]	2.378 [1.777-2.925]
COA-PWY-1: superpathway of	0	negligible	1.959 [0.000-2.836]	2.179 [1.205-2.884]	2.199 [1.513-2.630]

coenzyme A biosynthesis III (mammals)					
COA-PWY: coenzyme A biosynthesis I (prokaryotic)	0	negligible	1.924 [1.135-2.833]	2.201 [1.325-2.885]	2.209 [1.535-2.618]
COBALSYN-PWY: superpathway of adenosylcobalamin salvage from cobinamide I	0.01	small	0.000 [0.000-0.000]	0.000 [0.000-0.000]	0.000 [0.000-0.000]
COLANSYN-PWY: colanic acid building blocks biosynthesis	0	negligible	0.000 [0.000-0.000]	0.000 [0.000-0.000]	0.000 [0.000-0.000]
COMPLETE-ARO-PWY: superpathway of aromatic amino acid biosynthesis	0	negligible	2.124 [0.000-2.724]	2.074 [0.413-2.746]	2.076 [0.920-2.517]
DAPLYSINESYN-PWY: L-lysine biosynthesis I	0.055	small	0.000 [0.000-1.558]	0.000 [0.000-0.000]	0.000 [0.000-1.361]
DTDPRHAMSYN-PWY: dTDP-β-L-rhamnose biosynthesis	0.05	small	0.000 [0.000-0.000]	0.000 [0.000-0.000]	0.000 [0.000-0.000]
FAO-PWY: fatty acid β-oxidation I (generic)	0	negligible	0.000 [0.000-0.000]	0.000 [0.000-0.000]	0.000 [0.000-0.000]
FASYN-ELONG-PWY: fatty acid elongation -- saturated	0	negligible	2.307 [0.626-2.883]	2.155 [1.218-2.822]	2.170 [1.445-2.650]
FERMENTATION-PWY: mixed acid fermentation	0	negligible	2.205 [0.763-2.873]	1.788 [0.262-2.783]	2.022 [1.455-2.705]
FOLSYN-PWY: superpathway of tetrahydrofolate biosynthesis and salvage	0	negligible	1.503 [0.000-2.299]	1.176 [0.000-2.471]	1.981 [0.000-2.468]
GALACTARDEG-PWY: D-galactarate degradation I	0	negligible	0.000 [0.000-0.000]	0.000 [0.000-0.000]	0.000 [0.000-0.000]

GALACTUROCAT-PWY: D-galacturonate degradation I	0	negligible	0.000 [0.000-0.000]	0.000 [0.000-0.000]	0.000 [0.000-0.000]
GLCMANNANAUT-PWY: superpathway of N-acetylglucosamine, N-acetylmannosamine and N-acetylneuraminate degradation	0	negligible	1.863 [0.758-2.773]	1.864 [0.305-2.829]	2.091 [1.475-2.703]
GLUCARDEG-PWY: D-glucarate degradation I	0	negligible	0.000 [0.000-0.000]	0.000 [0.000-0.000]	0.000 [0.000-0.000]
GLUCARGALACTSUPER-PWY: superpathway of D-glucarate and D-galactarate degradation	0	negligible	0.000 [0.000-0.000]	0.000 [0.000-0.000]	0.000 [0.000-0.000]
GLUCONEO-PWY: gluconeogenesis I	0	negligible	0.000 [0.000-1.999]	0.000 [0.000-0.000]	0.000 [0.000-1.875]
GLUCOSE1PMETAB-PWY: glucose and glucose-1-phosphate degradation	0	negligible	0.000 [0.000-0.000]	0.000 [0.000-0.000]	0.000 [0.000-0.000]
GLUCUROCAT-PWY: superpathway of β-D-glucuronosides degradation	0	negligible	0.000 [0.000-0.000]	0.000 [0.000-0.000]	0.000 [0.000-0.000]
GLUTORN-PWY: L-ornithine biosynthesis I	0	negligible	2.000 [0.874-2.592]	1.754 [0.409-2.413]	1.873 [1.288-2.454]
GLYCOCAT-PWY: glycogen degradation I	0	negligible	0.000 [0.000-0.000]	0.000 [0.000-0.000]	0.000 [0.000-0.000]
GLYCOGENSYNTH-PWY: glycogen biosynthesis I (from ADP-D-Glucose)	0	negligible	0.000 [0.000-0.308]	0.000 [0.000-0.000]	0.000 [0.000-0.000]

GLYCOLYSIS-E-D: superpathway of glycolysis and the Entner-Doudoroff pathway	0	negligible	0.000 [0.000-0.000]	0.000 [0.000-0.000]	0.000 [0.000-0.000]
GLYCOLYSIS: glycolysis I (from glucose 6-phosphate)	0	negligible	2.313 [0.000-2.851]	2.252 [0.490-2.808]	2.114 [1.325-2.697]
GLYOXYLATE-BYPASS: glyoxylate cycle	0	negligible	0.000 [0.000-0.000]	0.000 [0.000-0.000]	0.000 [0.000-0.000]
HEME-BIOSYNTHESIS-II-1: heme b biosynthesis V (aerobic)	0	negligible	0.000 [0.000-0.000]	0.000 [0.000-0.000]	0.000 [0.000-0.000]
HEME-BIOSYNTHESIS-II: heme b biosynthesis I (aerobic)	0	negligible	1.942 [1.107-3.029]	2.202 [0.424-3.076]	2.250 [1.618-2.865]
HEMESYN2-PWY: heme b biosynthesis II (oxygen-independent)	0	negligible	1.927 [1.017-2.237]	1.152 [0.000-2.651]	1.883 [1.107-2.287]
HISDEG-PWY: L-histidine degradation I	0	negligible	2.005 [1.289-2.810]	2.263 [1.331-2.968]	2.335 [1.577-2.892]
HISTSYN-PWY: L-histidine biosynthesis	0	negligible	2.259 [0.752-2.775]	1.266 [1.014-1.491]	1.861 [1.306-2.676]
HOMOSER-METSYN-PWY: L-methionine biosynthesis I	0	negligible	0.000 [0.000-0.345]	0.000 [0.000-0.000]	0.000 [0.000-0.000]
HSERMETANA-PWY: L-methionine biosynthesis III	0	negligible	1.864 [0.849-2.213]	1.733 [1.381-2.054]	1.986 [1.346-2.630]
ILEUSYN-PWY: L-isoleucine biosynthesis I (from threonine)	0	negligible	2.565 [1.316-3.085]	2.302 [1.593-2.996]	2.452 [1.642-2.894]
LACTOSECAT-PWY: lactose and galactose degradation I	0	negligible	2.108 [1.040-2.957]	2.382 [1.525-3.036]	2.382 [1.737-2.941]

LIPASYN-PWY: phospholipases	0.012	small	0.000 [0.000-0.733]	0.000 [0.000-0.000]	0.000 [0.000-0.000]
MET-SAM-PWY: superpathway of S-adenosyl-L-methionine biosynthesis	0	negligible	0.000 [0.000-0.414]	0.000 [0.000-0.000]	0.000 [0.000-0.000]
METHGLYUT-PWY: superpathway of methylglyoxal degradation	0	negligible	0.728 [0.000-2.143]	0.880 [0.000-2.271]	0.000 [0.000-2.135]
METSYN-PWY: superpathway of L-homoserine and L-methionine biosynthesis	0	negligible	0.000 [0.000-0.402]	0.000 [0.000-0.000]	0.000 [0.000-0.000]
NAGLIPASYN-PWY: lipid IVA biosynthesis (E. coli)	0	negligible	0.000 [0.000-0.000]	0.000 [0.000-0.000]	0.000 [0.000-0.000]
NONMEVIPP-PWY: methylerythritol phosphate pathway I	0	negligible	0.000 [0.000-0.289]	0.000 [0.000-0.000]	0.000 [0.000-0.000]
NONOXIPENT-PWY: pentose phosphate pathway (non-oxidative branch) I	0	negligible	2.614 [1.001-3.179]	2.227 [1.589-3.117]	2.472 [1.906-2.963]
P101-PWY: ectoine biosynthesis	0	negligible	0.000 [0.000-0.000]	0.000 [0.000-0.000]	0.000 [0.000-0.000]
P105-PWY: TCA cycle IV (2-oxoglutarate decarboxylase)	0	negligible	0.000 [0.000-0.320]	0.000 [0.000-0.000]	0.000 [0.000-0.000]
P108-PWY: pyruvate fermentation to propanoate I	0.05	small	0.000 [0.000-0.000]	0.000 [0.000-0.000]	0.000 [0.000-0.000]
P125-PWY: superpathway of (R,R)-butanediol biosynthesis	0	negligible	2.074 [1.275-3.070]	2.216 [1.266-3.132]	2.290 [1.890-3.032]
P161-PWY: acetylene	0	negligible	0.000 [0.000-0.310]	0.000 [0.000-0.000]	0.000 [0.000-0.000]

degradation (anaerobic)					
P185-PWY: formaldehyde assimilation III (dihydroxyacetone cycle)	0	negligible	0.000 [0.000-0.000]	0.000 [0.000-0.000]	0.000 [0.000-0.000]
P221-PWY: octane oxidation	0	negligible	1.578 [1.195-2.657]	2.051 [0.368-2.797]	2.205 [1.615-2.767]
P23-PWY: reductive TCA cycle I	0.05	small	0.000 [0.000-0.000]	0.000 [0.000-0.000]	0.000 [0.000-0.000]
P4-PWY: superpathway of L-lysine, L-threonine and L-methionine biosynthesis I	0	negligible	0.000 [0.000-0.000]	0.000 [0.000-0.000]	0.000 [0.000-0.000]
P41-PWY: pyruvate fermentation to acetate and (S)-lactate I	0	negligible	2.312 [1.226-3.022]	2.381 [1.759-3.068]	2.536 [1.262-3.089]
P42-PWY: incomplete reductive TCA cycle	0	negligible	0.000 [0.000-0.409]	0.000 [0.000-0.000]	0.000 [0.000-1.491]
P441-PWY: superpathway of N-acetylneuraminate degradation	0	negligible	0.000 [0.000-0.416]	0.000 [0.000-0.000]	0.000 [0.000-0.000]
P461-PWY: hexitol fermentation to lactate, formate, ethanol and acetate	0	negligible	0.000 [0.000-0.335]	0.000 [0.000-0.000]	0.000 [0.000-0.000]
P562-PWY: myo-inositol degradation I	0.01	small	0.000 [0.000-0.000]	0.000 [0.000-0.000]	0.000 [0.000-0.000]
PANTO-PWY: phosphopantothenate biosynthesis I	0	negligible	2.495 [1.232-2.906]	2.384 [1.450-3.031]	2.490 [1.640-2.920]
PANTOSYN-PWY: superpathway of coenzyme A	0	negligible	2.091 [1.093-2.868]	2.267 [1.394-2.980]	2.329 [1.590-2.764]

biosynthesis I (bacteria)					
PENTOSE-P-PWY: pentose phosphate pathway	0	negligible	2.453 [0.810-3.016]	2.182 [1.407-2.977]	2.290 [1.713-2.826]
PEPTIDOGLYCANSYN-PWY: peptidoglycan biosynthesis I (meso-diaminopimelate containing)	0	negligible	2.311 [0.747-2.899]	2.285 [1.239-2.958]	2.262 [1.594-2.782]
PHOSLIPSYN-PWY: superpathway of phospholipid biosynthesis I (bacteria)	0	negligible	0.000 [0.000-0.000]	0.000 [0.000-0.000]	0.000 [0.000-0.000]
POLYAMSYN-PWY: superpathway of polyamine biosynthesis I	0	negligible	0.000 [0.000-0.000]	0.000 [0.000-0.000]	0.000 [0.000-0.000]
POLYISOPRENSYN-PWY: polyisoprenoid biosynthesis (E. coli)	0	negligible	1.800 [0.800-2.850]	1.872 [1.131-2.903]	2.199 [1.633-2.670]
PPGPPMET-PWY: ppGpp metabolism	0	negligible	0.000 [0.000-0.000]	0.000 [0.000-0.000]	0.000 [0.000-0.000]
PWY-1042: glycolysis IV	0	negligible	2.513 [1.259-3.054]	2.399 [1.643-3.104]	2.383 [1.810-3.067]
PWY-1269: CMP-3-deoxy-D-manno-octulosonate biosynthesis	0	negligible	0.000 [0.000-0.000]	0.000 [0.000-0.000]	0.000 [0.000-0.000]
PWY-1861: formaldehyde assimilation II (assimilatory RuMP Cycle)	0	negligible	2.281 [0.857-3.025]	2.281 [1.493-3.062]	2.375 [1.802-2.930]
PWY-241: C4 photosynthetic carbon assimilation cycle, NADP-ME type	0.05	small	0.000 [0.000-0.000]	0.000 [0.000-0.000]	0.000 [0.000-0.000]

PWY-2941: L-lysine biosynthesis II	0	negligible	1.991 [1.024-2.760]	1.903 [0.304-2.857]	2.246 [1.406-2.788]
PWY-2942: L-lysine biosynthesis III	0	negligible	2.022 [1.140-2.794]	2.149 [1.314-2.872]	2.250 [1.318-2.826]
PWY-3001: superpathway of L-isoleucine biosynthesis I	0	negligible	2.385 [0.000-2.863]	2.240 [0.457-2.908]	2.351 [1.440-2.802]
PWY-3801: sucrose degradation II (sucrose synthase)	0.056	small	0.000 [0.000-0.391]	0.000 [0.000-0.000]	0.000 [0.000-0.000]
PWY-3841: folate transformations II (plants)	0	negligible	2.181 [1.046-2.765]	2.206 [1.321-2.691]	2.165 [1.264-2.580]
PWY-4041: γ-glutamyl cycle	0.056	small	0.000 [0.000-0.206]	0.000 [0.000-0.000]	0.000 [0.000-0.000]
PWY-4984: urea cycle	0	negligible	2.164 [0.971-2.778]	2.201 [1.404-2.893]	2.309 [1.154-2.598]
PWY-5005: biotin biosynthesis II	0	negligible	1.768 [0.740-2.772]	1.677 [0.210-2.793]	1.992 [0.000-2.682]
PWY-5022: 4-aminobutanoate degradation V	0.133	moderate	0.000 [0.000-0.000]	0.000 [0.000-0.000]	1.975 [0.000-2.575]
PWY-5028: L-histidine degradation II	0	negligible	0.000 [0.000-0.000]	0.000 [0.000-0.000]	0.000 [0.000-0.000]
PWY-5030: L-histidine degradation III	0.05	small	0.000 [0.000-0.000]	0.000 [0.000-0.000]	0.000 [0.000-0.000]
PWY-5097: L-lysine biosynthesis VI	0	negligible	2.155 [0.997-2.728]	1.890 [0.301-2.849]	2.237 [1.364-2.799]
PWY-5100: pyruvate fermentation to acetate and lactate II	0	negligible	2.312 [1.374-3.022]	2.381 [1.759-3.068]	2.536 [1.524-3.089]
PWY-5103: L-isoleucine biosynthesis III	0	negligible	2.493 [1.274-3.019]	2.237 [1.549-2.908]	2.379 [1.553-2.828]
PWY-5121: superpathway of geranylgeranyl diphosphate biosynthesis II (via MEP)	0	negligible	0.000 [0.000-0.324]	0.000 [0.000-0.000]	0.000 [0.000-0.000]

PWY-5136: fatty acid β-oxidation II (plant peroxisome)	0	negligible	0.000 [0.000-0.225]	0.000 [0.000-0.000]	0.000 [0.000-0.000]
PWY-5138: fatty acid β-oxidation IV (unsaturated, even number)	0	negligible	0.000 [0.000-0.000]	0.000 [0.000-0.000]	0.000 [0.000-0.000]
PWY-5154: L-arginine biosynthesis III (via N-acetyl-L-citrulline)	0	negligible	0.000 [0.000-0.479]	0.000 [0.000-0.000]	0.000 [0.000-0.000]
PWY-5188: tetrapyrrole biosynthesis I (from glutamate)	0	negligible	2.316 [0.830-2.898]	2.298 [1.243-3.039]	2.302 [1.621-2.848]
PWY-5189: tetrapyrrole biosynthesis II (from glycine)	0	negligible	1.748 [0.720-2.467]	1.960 [0.323-2.734]	2.127 [1.068-2.456]
PWY-5265: peptidoglycan biosynthesis II (staphylococci)	0	negligible	2.080 [0.000-2.863]	2.208 [0.449-2.841]	2.207 [0.000-2.693]
PWY-5345: superpathway of L-methionine biosynthesis (by sulfhydrylation)	0	negligible	0.000 [0.000-1.715]	0.000 [0.000-0.000]	0.000 [0.000-1.274]
PWY-5347: superpathway of L-methionine biosynthesis (transsulfuration)	0	negligible	0.000 [0.000-0.415]	0.000 [0.000-0.000]	0.000 [0.000-0.000]
PWY-5367: petroselinate biosynthesis	0.05	small	0.000 [0.000-0.000]	0.000 [0.000-0.000]	0.000 [0.000-0.000]
PWY-5384: sucrose degradation IV (sucrose phosphorylase)	0	negligible	2.439 [0.000-2.964]	1.237 [0.000-2.807]	2.277 [1.525-2.695]
PWY-5484: glycolysis II (from fructose 6-phosphate)	0	negligible	2.231 [0.762-2.776]	2.233 [0.486-2.749]	2.034 [1.277-2.618]
PWY-5505: L-glutamate and L-	0	negligible	0.000 [0.000-0.000]	0.000 [0.000-0.000]	0.000 [0.000-0.000]

glutamine biosynthesis					
PWY-561: superpathway of glyoxylate cycle and fatty acid degradation	0	negligible	0.000 [0.000-0.000]	0.000 [0.000-0.000]	0.000 [0.000-0.000]
PWY-5659: GDP-mannose biosynthesis	0	negligible	0.000 [0.000-0.211]	0.000 [0.000-0.000]	0.000 [0.000-0.000]
PWY-5667: CDP-diacylglycerol biosynthesis I	0	negligible	2.302 [0.000-2.839]	1.228 [0.000-2.734]	2.087 [1.396-2.613]
PWY-5675: nitrate reduction V (assimilatory)	0	negligible	0.000 [0.000-0.000]	0.000 [0.000-0.000]	0.000 [0.000-0.000]
PWY-5676: acetyl-CoA fermentation to butanoate II	0.05	small	0.000 [0.000-0.000]	0.000 [0.000-0.000]	0.000 [0.000-0.000]
PWY-5686: UMP biosynthesis I	0	negligible	2.434 [0.878-2.969]	2.276 [1.387-2.922]	2.407 [1.673-2.769]
PWY-5690: TCA cycle II (plants and fungi)	0	negligible	0.000 [0.000-0.000]	0.000 [0.000-0.000]	0.000 [0.000-0.000]
PWY-5695: inosine 5'-phosphate degradation	0	negligible	2.655 [1.375-3.263]	2.238 [1.454-3.166]	2.545 [1.922-3.068]
PWY-5723: Rubisco shunt	0	negligible	0.000 [0.000-0.000]	0.000 [0.000-0.000]	0.000 [0.000-0.000]
PWY-5837: 2-carboxy-1,4-naphthoquinol biosynthesis	0	negligible	1.559 [0.000-2.743]	1.789 [0.285-2.777]	2.103 [1.262-2.613]
PWY-5838: superpathway of menaquinol-8 biosynthesis I	0	negligible	0.000 [0.000-0.000]	0.000 [0.000-0.000]	0.000 [0.000-0.000]
PWY-5840: superpathway of menaquinol-7 biosynthesis	0	negligible	1.670 [0.000-2.759]	1.779 [0.269-2.761]	2.112 [1.395-2.698]
PWY-5855: ubiquinol-7 biosynthesis (early decarboxylation)	0	negligible	0.000 [0.000-0.000]	0.000 [0.000-0.000]	0.000 [0.000-0.000]
PWY-5861: superpathway of demethylmenaqui	0	negligible	0.000 [0.000-0.000]	0.000 [0.000-0.000]	0.000 [0.000-0.000]

nol-8 biosynthesis I					
PWY-5897: superpathway of menaquinol-11 biosynthesis	0	negligible	1.665 [0.000-2.757]	1.784 [0.272-2.761]	2.115 [1.382-2.694]
PWY-5898: superpathway of menaquinol-12 biosynthesis	0	negligible	1.665 [0.000-2.757]	1.784 [0.272-2.761]	2.115 [1.382-2.694]
PWY-5899: superpathway of menaquinol-13 biosynthesis	0	negligible	1.665 [0.000-2.757]	1.784 [0.272-2.761]	2.115 [1.382-2.694]
PWY-5910: superpathway of geranylgeranyldiphosphate biosynthesis I (via mevalonate)	0	negligible	1.592 [0.780-2.179]	1.213 [0.000-2.696]	1.968 [1.479-2.602]
PWY-5913: partial TCA cycle (obligate autotrophs)	0.038	small	0.000 [0.000-1.225]	0.000 [0.000-0.000]	0.000 [0.000-0.000]
PWY-5918: superpathway of heme b biosynthesis from glutamate	0	negligible	1.825 [0.000-2.709]	2.113 [0.382-2.831]	2.096 [1.307-2.659]
PWY-5920: superpathway of heme b biosynthesis from glycine	0	negligible	0.000 [0.000-0.000]	0.000 [0.000-2.021]	0.000 [0.000-0.000]
PWY-5941: glycogen degradation II	0	negligible	0.000 [0.000-0.000]	0.000 [0.000-0.000]	0.000 [0.000-0.000]
PWY-5971: palmitate biosynthesis (type II fatty acid synthase)	0.05	small	0.000 [0.000-0.000]	0.000 [0.000-0.000]	0.000 [0.000-0.000]
PWY-5973: cis-vaccenate biosynthesis	0	negligible	0.000 [0.000-0.296]	0.000 [0.000-1.231]	0.000 [0.000-1.329]
PWY-5981: CDP-diacylglycerol biosynthesis III	0	negligible	1.733 [0.828-2.660]	1.924 [0.354-2.713]	2.121 [1.273-2.510]
PWY-5989: stearate	0	negligible	0.000 [0.000-0.315]	0.000 [0.000-0.000]	0.000 [0.000-1.403]

biosynthesis II (bacteria and plants)					
PWY-6084: 3,5-dichlorocatechol degradation	0.05	small	0.000 [0.000-0.000]	0.000 [0.000-0.000]	0.000 [0.000-0.000]
PWY-6121: 5-aminoimidazole ribonucleotide biosynthesis I	0	negligible	2.364 [1.262-2.957]	2.258 [1.554-2.923]	2.303 [1.533-2.848]
PWY-6122: 5-aminoimidazole ribonucleotide biosynthesis II	0	negligible	2.467 [1.364-3.059]	2.297 [1.562-2.982]	2.409 [1.580-2.944]
PWY-6123: inosine-5'-phosphate biosynthesis I	0	negligible	2.257 [1.086-2.876]	2.202 [1.347-2.819]	2.118 [1.468-2.709]
PWY-6124: inosine-5'-phosphate biosynthesis II	0	negligible	2.229 [1.067-2.863]	2.236 [1.374-2.836]	2.132 [1.501-2.673]
PWY-6125: superpathway of guanosine nucleotides de novo biosynthesis II	0	negligible	2.352 [0.765-2.935]	2.290 [1.428-2.892]	2.231 [1.356-2.615]
PWY-6126: superpathway of adenosine nucleotides de novo biosynthesis II	0	negligible	2.259 [0.753-2.832]	2.248 [1.412-2.812]	2.164 [1.386-2.598]
PWY-6147: 6-hydroxymethyl-dihydropterin diphosphate biosynthesis I	0	negligible	1.802 [1.106-2.702]	1.940 [1.182-2.788]	2.186 [1.390-2.638]
PWY-6151: S-adenosyl-L-methionine salvage I	0	negligible	2.452 [0.835-2.987]	2.250 [1.430-2.928]	2.376 [1.515-2.771]
PWY-6163: chorismate biosynthesis from 3-dehydroquinate	0	negligible	2.047 [0.000-2.740]	2.130 [0.437-2.817]	2.057 [1.475-2.470]
PWY-6168: flavin biosynthesis III (fungi)	0.01	small	0.000 [0.000-0.000]	0.000 [0.000-0.000]	0.000 [0.000-0.000]

PWY-621: sucrose degradation III (sucrose invertase)	0	negligible	2.383 [0.000-2.889]	1.210 [0.000-2.749]	2.222 [1.451-2.677]
PWY-6270: isoprene biosynthesis I	0	negligible	0.000 [0.000-0.257]	0.000 [0.000-0.000]	0.000 [0.000-0.000]
PWY-6277: superpathway of 5-aminoimidazole ribonucleotide biosynthesis	0	negligible	2.467 [1.364-3.059]	2.297 [1.562-2.982]	2.409 [1.580-2.944]
PWY-6282: palmitoleate biosynthesis I (from (5Z)-dodec-5-enoate)	0	negligible	0.000 [0.000-0.315]	0.000 [0.000-0.000]	0.000 [0.000-1.330]
PWY-6284: superpathway of unsaturated fatty acids biosynthesis (E. coli)	0.05	small	0.000 [0.000-0.000]	0.000 [0.000-0.000]	0.000 [0.000-0.000]
PWY-6292: superpathway of L-cysteine biosynthesis (mammalian)	0	negligible	0.000 [0.000-0.000]	0.000 [0.000-0.000]	0.000 [0.000-0.000]
PWY-6293: superpathway of L-cysteine biosynthesis (fungi)	0	negligible	0.000 [0.000-0.000]	0.000 [0.000-0.000]	0.000 [0.000-0.000]
PWY-6305: superpathway of putrescine biosynthesis	0.05	small	0.000 [0.000-0.000]	0.000 [0.000-0.000]	0.000 [0.000-1.040]
PWY-6317: D-galactose degradation I (Leloir pathway)	0.038	small	0.000 [0.000-1.401]	0.000 [0.000-0.000]	0.000 [0.000-0.000]
PWY-6385: peptidoglycan biosynthesis III (mycobacteria)	0	negligible	2.319 [0.719-2.921]	2.244 [1.255-2.975]	2.299 [1.611-2.805]
PWY-6386: UDP-N-acetylmuramoyl-pentapeptide biosynthesis II	0	negligible	2.127 [0.974-2.870]	2.328 [1.211-2.933]	2.224 [1.570-2.772]

(lysine-containing)					
PWY-6387: UDP-N-acetylmuramoyl-pentapeptide biosynthesis I (meso-diaminopimelate containing)	0	negligible	2.285 [1.007-2.901]	2.319 [1.246-2.972]	2.273 [1.629-2.803]
PWY-6421: arsenate detoxification IV (mycothiol)	0.05	small	0.000 [0.000-0.000]	0.000 [0.000-0.000]	0.000 [0.000-0.000]
PWY-6470: peptidoglycan biosynthesis V (β-lactam resistance)	0.05	small	0.000 [0.000-0.000]	0.000 [0.000-0.000]	0.000 [0.000-0.000]
PWY-6507: 4-deoxy-L-threo-hex-4-enopyranuronate degradation	0	negligible	0.000 [0.000-0.000]	0.000 [0.000-0.000]	0.000 [0.000-0.000]
PWY-6519: 8-amino-7-oxononanoate biosynthesis I	0	negligible	0.000 [0.000-0.000]	0.000 [0.000-0.000]	0.000 [0.000-0.000]
PWY-6545: pyrimidine deoxyribonucleotides de novo biosynthesis III	0	negligible	0.000 [0.000-0.000]	0.000 [0.000-0.000]	0.000 [0.000-0.000]
PWY-6549: L-glutamine biosynthesis III	0	negligible	0.000 [0.000-0.000]	0.000 [0.000-0.000]	0.000 [0.000-0.000]
PWY-6606: guanosine nucleotides degradation II	0	negligible	0.000 [0.000-0.247]	0.000 [0.000-0.000]	0.000 [0.000-0.000]
PWY-6607: guanosine nucleotides degradation I	0	negligible	0.000 [0.000-0.000]	0.000 [0.000-0.000]	0.000 [0.000-0.000]
PWY-6608: guanosine nucleotides degradation III	0	negligible	0.000 [0.000-0.259]	0.000 [0.000-0.000]	0.000 [0.000-0.000]
PWY-6609: adenine and	0	negligible	2.488 [1.028-2.992]	2.262 [1.481-2.915]	2.272 [1.541-2.546]

adenosine salvage III					
PWY-6612: superpathway of tetrahydrofolate biosynthesis	0	negligible	1.478 [0.000-2.209]	1.117 [0.000-2.519]	1.954 [0.000-2.418]
PWY-6628: superpathway of L-phenylalanine biosynthesis	0	negligible	2.069 [0.000-2.681]	1.985 [0.383-2.678]	2.021 [0.777-2.480]
PWY-6630: superpathway of L-tyrosine biosynthesis	0	negligible	1.042 [0.000-2.261]	1.790 [0.391-2.075]	1.682 [0.000-2.078]
PWY-6700: queuosine biosynthesis I (de novo)	0	negligible	2.428 [1.279-2.961]	2.261 [1.482-2.949]	2.273 [1.502-2.736]
PWY-6703: preQ0 biosynthesis	0	negligible	2.274 [1.134-2.857]	2.265 [1.379-2.798]	2.104 [1.557-2.633]
PWY-6823: molybdopterin biosynthesis	0	negligible	2.212 [1.107-2.774]	2.141 [1.428-2.702]	2.124 [1.520-2.532]
PWY-6859: all-trans-farnesol biosynthesis	0	negligible	1.689 [0.721-2.833]	1.782 [0.945-2.841]	2.115 [1.568-2.703]
PWY-6897: thiamine diphosphate salvage II	0	negligible	1.731 [0.000-2.399]	1.783 [1.128-2.439]	1.885 [1.140-2.290]
PWY-6901: superpathway of glucose and xylose degradation	0	negligible	1.893 [0.000-2.580]	1.764 [0.309-2.584]	1.864 [1.375-2.649]
PWY-6922: L-Nδ-acetylornithine biosynthesis	0	negligible	0.000 [0.000-0.354]	0.000 [0.000-0.000]	0.000 [0.000-0.000]
PWY-6936: seleno-amino acid biosynthesis (plants)	0	negligible	2.409 [1.038-2.971]	2.366 [1.549-2.934]	2.244 [1.317-2.872]
PWY-6969: TCA cycle V (2-oxoglutarate synthase)	0.118	moderate	0.693 [0.000-1.750]	0.000 [0.000-0.000]	0.000 [0.000-1.464]
PWY-702: L-methionine biosynthesis II	0	negligible	2.368 [0.616-2.991]	2.198 [0.444-2.923]	2.310 [1.455-2.880]

PWY-7111: pyruvate fermentation to isobutanol (engineered)	0	negligible	2.484 [1.362-2.973]	2.235 [1.321-2.892]	2.333 [1.576-2.836]
PWY-7115: C4 photosynthetic carbon assimilation cycle, NAD-ME type	0	negligible	0.000 [0.000-0.000]	0.000 [0.000-0.000]	0.000 [0.000-0.000]
PWY-7117: C4 photosynthetic carbon assimilation cycle, PEPCK type	0	negligible	0.000 [0.000-0.000]	0.000 [0.000-0.000]	0.000 [0.000-0.000]
PWY-7118: chitin deacetylation	0	negligible	0.000 [0.000-0.000]	0.000 [0.000-0.000]	0.000 [0.000-0.000]
PWY-7184: pyrimidine deoxyribonucleotides de novo biosynthesis I	0.056	small	0.000 [0.000-0.378]	0.000 [0.000-0.000]	0.000 [0.000-0.000]
PWY-7197: pyrimidine deoxyribonucleotide phosphorylation	0	negligible	1.942 [0.692-2.716]	2.177 [1.263-2.753]	2.037 [1.224-2.433]
PWY-7198: pyrimidine deoxyribonucleotides de novo biosynthesis IV	0	negligible	0.000 [0.000-1.405]	0.000 [0.000-0.000]	0.000 [0.000-0.000]
PWY-7199: pyrimidine deoxyribonucleosides salvage	0	negligible	1.819 [1.321-2.664]	2.240 [0.496-2.688]	2.180 [1.533-2.416]
PWY-7200: superpathway of pyrimidine deoxyribonucleoside salvage	0	negligible	1.253 [0.000-2.529]	2.046 [0.449-2.529]	1.964 [1.273-2.353]
PWY-7204: pyridoxal 5'-phosphate salvage II (plants)	0.05	small	0.000 [0.000-0.000]	0.000 [0.000-0.000]	0.000 [0.000-0.000]
PWY-7208: superpathway of pyrimidine nucleobases salvage	0	negligible	2.351 [0.716-2.883]	2.258 [1.182-2.873]	2.186 [1.359-2.558]

PWY-7210: pyrimidine deoxyribonucleotides biosynthesis from CTP	0.056	small	0.000 [0.000-0.389]	0.000 [0.000-0.000]	0.000 [0.000-0.000]
PWY-7211: superpathway of pyrimidine deoxyribonucleotides de novo biosynthesis	0	negligible	0.000 [0.000-0.000]	0.000 [0.000-0.000]	0.000 [0.000-0.000]
PWY-7220: adenosine deoxyribonucleotides de novo biosynthesis II	0	negligible	2.311 [0.738-2.919]	2.252 [1.350-2.857]	2.143 [1.281-2.518]
PWY-7221: guanosine ribonucleotides de novo biosynthesis	0	negligible	2.390 [1.035-3.058]	2.369 [1.645-2.953]	2.403 [1.485-2.856]
PWY-7222: guanosine deoxyribonucleotides de novo biosynthesis II	0	negligible	2.311 [0.738-2.919]	2.252 [1.350-2.857]	2.143 [1.281-2.518]
PWY-7228: superpathway of guanosine nucleotides de novo biosynthesis I	0	negligible	2.388 [0.795-2.977]	2.318 [1.475-2.924]	2.275 [1.402-2.672]
PWY-7229: superpathway of adenosine nucleotides de novo biosynthesis I	0	negligible	2.269 [0.772-2.848]	2.267 [1.453-2.825]	2.190 [1.441-2.650]
PWY-7234: inosine-5'-phosphate biosynthesis III	0	negligible	2.187 [0.966-2.829]	2.154 [1.177-2.749]	2.080 [1.382-2.641]
PWY-7237: myo-, chiro- and scyllo-inositol degradation	0.01	small	0.000 [0.000-0.000]	0.000 [0.000-0.000]	0.000 [0.000-0.000]
PWY-7238: sucrose biosynthesis II	0	negligible	0.000 [0.000-0.000]	0.000 [0.000-0.000]	0.000 [0.000-0.000]

PWY-7242: D-fructuronate degradation	0	negligible	0.000 [0.000-0.000]	0.000 [0.000-0.000]	0.000 [0.000-0.000]
PWY-724: superpathway of L-lysine, L-threonine and L-methionine biosynthesis II	0	negligible	2.135 [0.000-2.744]	1.282 [0.000-2.834]	2.244 [1.335-2.760]
PWY-7254: TCA cycle VII (acetate-producers)	0.028	small	0.000 [0.000-1.475]	0.000 [0.000-0.000]	0.000 [0.000-0.000]
PWY-7282: 4-amino-2-methyl-5-diphosphomethyl pyrimidine biosynthesis II	0	negligible	0.000 [0.000-0.392]	0.000 [0.000-0.000]	0.000 [0.000-1.283]
PWY-7323: superpathway of GDP-mannose-derived O-antigen building blocks biosynthesis	0	negligible	0.000 [0.000-0.000]	0.000 [0.000-0.000]	0.000 [0.000-0.000]
PWY-7328: superpathway of UDP-glucose-derived O-antigen building blocks biosynthesis	0	negligible	0.000 [0.000-0.000]	0.000 [0.000-0.000]	0.000 [0.000-0.000]
PWY-7345: superpathway of anaerobic sucrose degradation	0	negligible	2.029 [0.000-2.686]	1.267 [0.000-2.781]	2.080 [1.315-2.641]
PWY-7356: thiamine diphosphate salvage IV (yeast)	0	negligible	1.615 [0.000-2.710]	1.996 [0.366-2.796]	2.168 [1.390-2.675]
PWY-7357: thiamine phosphate formation from pyrithiamine and oxythiamine (yeast)	0	negligible	1.701 [0.000-2.433]	2.092 [1.274-2.500]	1.913 [1.184-2.429]
PWY-7383: anaerobic energy metabolism (invertebrates, cytosol)	0	negligible	0.000 [0.000-0.000]	0.000 [0.000-0.000]	0.000 [0.000-0.000]

PWY-7388: octanoyl-[acyl-carrier protein] biosynthesis (mitochondria, yeast)	0	negligible	1.389 [0.000-2.908]	1.942 [0.287-2.955]	2.166 [1.461-2.758]
PWY-7391: isoprene biosynthesis II (engineered)	0.201	large	0.000 [0.000-0.000]	0.000 [0.000-1.463]	1.516 [0.000-1.966]
PWY-7392: taxadiene biosynthesis (engineered)	0	negligible	0.000 [0.000-0.290]	0.000 [0.000-0.000]	0.000 [0.000-0.000]
PWY-7396: butanol and isobutanol biosynthesis (engineered)	0.074	moderate	0.000 [0.000-0.000]	0.000 [0.000-0.000]	0.000 [0.000-1.344]
PWY-7400: L-arginine biosynthesis IV (archaebacteria)	-0.102	moderate	0.000 [0.000-0.641]	0.000 [0.000-0.000]	0.000 [0.000-0.000]
PWY-7560: methylerythritol phosphate pathway II	0	negligible	0.000 [0.000-0.261]	0.000 [0.000-0.000]	0.000 [0.000-0.000]
PWY-7663: gondoate biosynthesis (anaerobic)	0	negligible	2.291 [0.492-2.918]	2.155 [1.134-2.880]	2.229 [1.445-2.754]
PWY-7664: oleate biosynthesis IV (anaerobic)	0	negligible	0.000 [0.000-0.330]	0.000 [0.000-0.000]	0.000 [0.000-1.360]
PWY-7790: UMP biosynthesis II	0	negligible	2.271 [0.878-2.830]	2.200 [1.353-2.919]	2.402 [1.664-2.768]
PWY-7791: UMP biosynthesis III	0	negligible	2.200 [0.806-2.813]	2.167 [1.245-2.823]	2.142 [1.546-2.680]
PWY-7851: coenzyme A biosynthesis II (eukaryotic)	0	negligible	1.912 [0.834-2.833]	2.201 [1.204-2.885]	2.209 [1.535-2.594]
PWY-7858: (5Z)-dodecenoate biosynthesis II	0	negligible	0.000 [0.000-0.000]	0.000 [0.000-0.000]	0.000 [0.000-0.000]
PWY-7942: 5-oxo-L-proline metabolism	0.05	small	0.000 [0.000-0.000]	0.000 [0.000-0.000]	0.000 [0.000-0.000]

PWY-7948: 4-oxopentanoate degradation	0.01	small	0.000 [0.000-0.000]	0.000 [0.000-0.000]	0.000 [0.000-0.000]
PWY-7953: UDP-N-acetylmuramoyl-pentapeptide biosynthesis III (meso-diaminopimelate containing)	0	negligible	2.161 [0.000-2.751]	1.217 [0.000-2.642]	2.091 [0.000-2.545]
PWY-7977: L-methionine biosynthesis IV	0	negligible	2.105 [1.035-2.639]	1.960 [1.210-2.669]	1.994 [1.221-2.554]
PWY-8004: Entner-Doudoroff pathway I	0	negligible	0.000 [0.000-0.379]	0.000 [0.000-0.000]	0.000 [0.000-0.000]
PWY-8011: L-serine biosynthesis II	0	negligible	0.000 [0.000-0.000]	0.000 [0.000-0.000]	0.000 [0.000-0.000]
PWY-801: homocysteine and cysteine interconversion	0	negligible	0.000 [0.000-0.000]	0.000 [0.000-0.000]	0.000 [0.000-0.000]
PWY-8073: lipid IVA biosynthesis (P. putida)	0	negligible	0.000 [0.000-0.000]	0.000 [0.000-0.000]	0.000 [0.000-0.000]
PWY-8131: 5'-deoxyadenosine degradation II	0	negligible	0.000 [0.000-0.000]	0.000 [0.000-0.000]	0.000 [0.000-0.000]
PWY-8178: pentose phosphate pathway (non-oxidative branch) II	0	negligible	2.509 [0.000-2.995]	2.187 [1.550-3.026]	2.275 [1.733-2.892]
PWY-8182: valproate β-oxidation	0.01	small	0.000 [0.000-0.000]	0.000 [0.000-0.000]	0.000 [0.000-0.000]
PWY-8187: L-arginine degradation XIII (reductive Stickland reaction)	0	negligible	2.024 [1.334-3.000]	2.423 [1.535-3.169]	2.314 [1.904-2.792]
PWY-821: superpathway of sulfur amino acid biosynthesis	0	negligible	0.000 [0.000-0.362]	0.000 [0.000-0.000]	0.000 [0.000-0.000]

(Saccharomyces cerevisiae)					
PWY-841: superpathway of purine nucleotides de novo biosynthesis I	0	negligible	2.301 [0.802-2.792]	2.256 [1.441-2.864]	2.207 [1.445-2.681]
PWY-922: mevalonate pathway I (eukaryotes and bacteria)	0	negligible	1.617 [0.839-2.088]	1.199 [0.000-2.703]	1.960 [1.456-2.652]
PWY-I9: L-cysteine biosynthesis VI (from L-methionine)	0	negligible	2.385 [0.618-2.869]	2.161 [1.233-2.796]	2.217 [1.471-2.672]
PWY0-1061: superpathway of L-alanine biosynthesis	0.056	small	0.000 [0.000-0.341]	0.000 [0.000-0.000]	0.000 [0.000-0.000]
PWY0-1241: ADP-L-glycero-β-D-manno-heptose biosynthesis	0	negligible	0.000 [0.000-0.000]	0.000 [0.000-0.000]	0.000 [0.000-0.000]
PWY0-1261: anhydromuropeptides recycling I	0	negligible	0.000 [0.000-0.000]	0.000 [0.000-0.000]	0.000 [0.000-0.000]
PWY0-1296: purine ribonucleosides degradation	0	negligible	2.674 [1.436-3.139]	2.349 [1.584-3.108]	2.431 [1.963-2.987]
PWY0-1297: superpathway of purine deoxyribonucleosides degradation	0	negligible	0.000 [0.000-0.382]	0.000 [0.000-0.000]	0.000 [0.000-0.941]
PWY0-1298: superpathway of pyrimidine deoxyribonucleosides degradation	0	negligible	0.000 [0.000-1.523]	0.000 [0.000-1.196]	0.000 [0.000-0.000]
PWY0-1319: CDP-diacylglycerol biosynthesis II	0	negligible	2.302 [0.000-2.839]	1.228 [0.000-2.734]	2.087 [1.396-2.613]
PWY0-1477: ethanolamine utilization	0	negligible	0.000 [0.000-0.310]	0.000 [0.000-0.000]	0.000 [0.000-0.000]

PWY0-1479: tRNA processing	0	negligible	0.000 [0.000-0.000]	0.000 [0.000-0.000]	0.000 [0.000-0.000]
PWY0-1586: peptidoglycan maturation (meso-diaminopimelate containing)	0	negligible	2.320 [0.000-2.918]	2.151 [1.244-2.879]	2.277 [1.582-2.698]
PWY0-162: superpathway of pyrimidine ribonucleotides de novo biosynthesis	0	negligible	2.263 [0.000-2.822]	2.190 [1.227-2.851]	2.177 [1.473-2.642]
PWY0-166: superpathway of pyrimidine deoxyribonucleotides de novo biosynthesis (E. coli)	0	negligible	0.000 [0.000-0.000]	0.000 [0.000-0.000]	0.000 [0.000-0.000]
PWY0-845: superpathway of pyridoxal 5'-phosphate biosynthesis and salvage	0	negligible	0.000 [0.000-0.000]	0.000 [0.000-0.000]	0.000 [0.000-0.000]
PWY0-862: (5Z)-dodecenoate biosynthesis I	0	negligible	0.000 [0.000-0.315]	0.000 [0.000-0.000]	0.000 [0.000-1.298]
PWY1G-0: mycothiol biosynthesis	0	negligible	0.000 [0.000-0.000]	0.000 [0.000-0.000]	0.000 [0.000-0.000]
PWY1ZNC-1: assimilatory sulfate reduction IV	0	negligible	0.471 [0.000-1.274]	0.000 [0.000-0.000]	0.000 [0.000-0.000]
PWY3O-4107: NAD salvage pathway V (PNC V cycle)	0.05	small	0.000 [0.000-0.000]	0.000 [0.000-0.000]	0.000 [0.000-1.182]
PWY4FS-7: phosphatidylglycerol biosynthesis I (plastidic)	0	negligible	0.000 [0.000-0.000]	0.000 [0.000-0.000]	0.000 [0.000-0.000]
PWY4FS-8: phosphatidylglycerol biosynthesis II (non-plastidic)	0	negligible	0.000 [0.000-0.000]	0.000 [0.000-0.000]	0.000 [0.000-0.000]

PWY4LZ-257: superpathway of fermentation (Chlamydomonas reinhardtii)	0	negligible	0.000 [0.000-0.401]	0.000 [0.000-0.000]	0.000 [0.000-0.000]
PWY66-389: phytol degradation	0	negligible	2.576 [1.506-3.101]	2.082 [0.283-3.233]	2.579 [1.964-3.105]
PWY66-391: fatty acid β-oxidation VI (mammalian peroxisome)	0	negligible	0.000 [0.000-0.000]	0.000 [0.000-0.000]	0.000 [0.000-0.000]
PWY66-399: gluconeogenesis III	0.05	small	0.000 [0.000-0.000]	0.000 [0.000-0.000]	0.000 [0.000-0.000]
PWY66-409: superpathway of purine nucleotide salvage	0	negligible	0.000 [0.000-0.484]	0.000 [0.000-0.000]	0.000 [0.000-0.000]
PWY66-429: fatty acid biosynthesis initiation (mitochondria)	0	negligible	2.188 [0.791-2.828]	2.123 [1.357-2.808]	2.093 [1.359-2.803]
PWY66-430: myristate biosynthesis (mitochondria)	0.05	small	0.000 [0.000-0.000]	0.000 [0.000-0.000]	0.000 [0.000-0.000]
PYRIDOXSYN-PWY: pyridoxal 5'-phosphate biosynthesis I	0	negligible	0.000 [0.000-0.000]	0.000 [0.000-0.000]	0.000 [0.000-0.000]
REDCITCYC: TCA cycle VI (Helicobacter)	0.074	moderate	0.000 [0.000-0.000]	0.000 [0.000-0.000]	0.000 [0.000-1.296]
RIBOSYN2-PWY: flavin biosynthesis I (bacteria and plants)	0	negligible	1.790 [0.000-2.601]	1.158 [0.000-2.559]	1.937 [1.273-2.481]
RUMP-PWY: formaldehyde oxidation I	0	negligible	1.340 [0.587-2.628]	1.077 [0.000-2.580]	1.900 [1.364-2.556]
SALVADEHYPOX-PWY: adenosine nucleotides degradation II	0	negligible	0.000 [0.000-0.000]	0.000 [0.000-0.000]	0.000 [0.000-0.000]
SER-GLYSYN-PWY:	0	negligible	2.173 [1.042-2.861]	2.223 [1.146-2.805]	2.235 [1.186-2.614]

superpathway of L-serine and glycine biosynthesis I					
SO4ASSIM-PWY: assimilatory sulfate reduction I	0	negligible	0.472 [0.000-1.478]	0.000 [0.000-0.000]	0.000 [0.000-0.999]
SULFATE-CYS-PWY: superpathway of sulfate assimilation and cysteine biosynthesis	0	negligible	0.000 [0.000-1.649]	0.000 [0.000-0.000]	0.000 [0.000-0.000]
TCA-GLYOX-BYPASS: superpathway of glyoxylate bypass and TCA	0	negligible	0.000 [0.000-0.000]	0.000 [0.000-0.000]	0.000 [0.000-0.000]
TCA: TCA cycle I (prokaryotic)	0	negligible	0.000 [0.000-0.000]	0.000 [0.000-0.000]	0.000 [0.000-0.000]
THISYNARA-PWY: superpathway of thiamine diphosphate biosynthesis III (eukaryotes)	0	negligible	0.000 [0.000-0.299]	0.000 [0.000-0.750]	0.000 [0.000-0.000]
THRESYN-PWY: superpathway of L-threonine biosynthesis	0	negligible	2.352 [0.000-2.906]	2.222 [0.451-2.867]	2.295 [1.365-2.756]
TRNA-CHARGING-PWY: tRNA charging	0	negligible	2.450 [0.802-2.892]	2.279 [0.462-2.977]	2.299 [1.565-2.848]
UDPNAGSYN-PWY: UDP-N-acetyl-D-glucosamine biosynthesis I	0	negligible	2.572 [1.315-2.999]	2.422 [1.571-3.075]	2.414 [1.858-2.940]
VALSYN-PWY: L-valine biosynthesis	0	negligible	2.649 [1.368-3.162]	2.376 [1.634-3.125]	2.520 [1.814-3.000]

Data are presented as median [IQR]. All values are rounded to three decimal places. The effect size (ϵ^2) was calculated using the Kruskal-Wallis test. Negative calculated adjusted effect sizes were constrained to 0, indicating a negligible effect.
