# Supplementary material for: Taxonomic and Functional Skin Microbiome Profiles in Nummular Eczema, Atopic Dermatitis, and Mixed Phenotypes: An Exploratory Shotgun Metagenomic Pilot Study
Source: Microorganisms. 2026 Jul 27;14(8):1631. doi: 10.3390/microorganisms14081631 (PMC13515936; doi:10.3390/microorganisms14081631)
Supplement: Supplementary file 1 [file microorganisms-14-01631-s001.zip › Supplementary Figure S1.pdf]

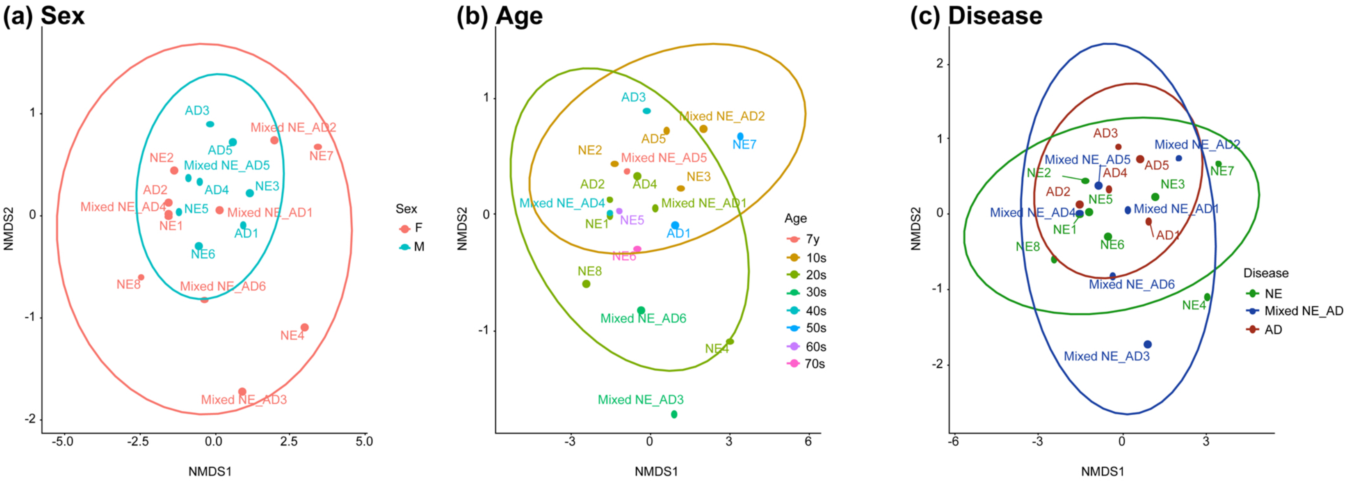

Figure S1. Beta diversity analysis by demographic and clinical variables.

Non-metric Multidimensional Scaling (NMDS) ordination plots based on Bray-Curtis dissimilarity, comparing the skin microbiome communities across groups. Samples are colored by (a) Sex, (b) Age, and (c) Disease phenotype (NE, Mixed NE_AD, and AD). While samples were not clearly clustered by age or sex, they showed partial ecological separation by diagnostic group. NE and AD samples occupied divergent positions in the ordination space, with the Mixed NE_AD samples consistently positioned between them, reflecting an overlapping phenotype.
